# Synthesis and physico-chemical properties of a H-cardanol triazole zinc porphyrin conjugate†

**DOI:** 10.1039/c8ra09998g

**Published:** 2019-02-05

**Authors:** H. Surya Prakash Rao, M. Kamalraj, M. Prabakaran

**Affiliations:** Department of Chemistry, Pondicherry University Pondicherry India hspr@yahoo.com hsp.rao@sharda.ac.in +914132654411 +919870414222; Sharda University Knowledge Park III Greater Noida Uttar Pradesh India 201306

## Abstract

Although a large number of natural and non-natural metalloporphyrins are known, examples with fluorescence and fat-soluble properties are rare. We have achieved the synthesis of a fluorescent and fat-soluble zinc porphyrin incorporating four units of hydrogenated cardanol (H-cardanol). The synthesis is sustainable since the product is derived from cashew-nut shell liquid (CNSL), which is a renewable and bio-waste material. The H-cardanol triazole zinc porphyrin conjugate (HTZPC) was synthesized through applying a copper(i) catalyzed azide–alkyne cycloaddition (CuAAC) reaction between a H-cardanol derived azide and a tetraarylporphyrin derived alkyne. The absorption and emission properties of the hydrocarbon solvent soluble HTZPC were evaluated using UV-vis and fluorescence emission spectra obtained in various solvents. The results were compared with related molecules like a triazole-zinc porphyrin conjugate (TZPC), zinc tetra-C(4)-methoxyphenyl porphyrin (ZP), and a H-cardanol-triazole conjugate (HTC). The results showed that HTZPC undergoes J-type aggregation in both non-polar and highly polar solvents, which is dictated by van der Waals attractive forces between H-cardanol units in polar solvents (*e.g.* methanol and dimethylformamide) and π–π stacking interactions between porphyrin units in non-polar solvents (hexane). Moreover, the spectra indicated that the triazole units could stabilize the zinc porphyrin *via* intermolecular coordinate-complex formation. We anticipate that fat-soluble HTZPC could find applications in medical fields (*e.g.* in the photodynamic therapy of fat tissue).

## Abstract 2

A fluorescent and fat-soluble zinc porphyrin incorporating four units of hydrogenated cardanol (H-cardanol) was synthesized, and its physico-chemical properties were characterized.

## Graphical abstract

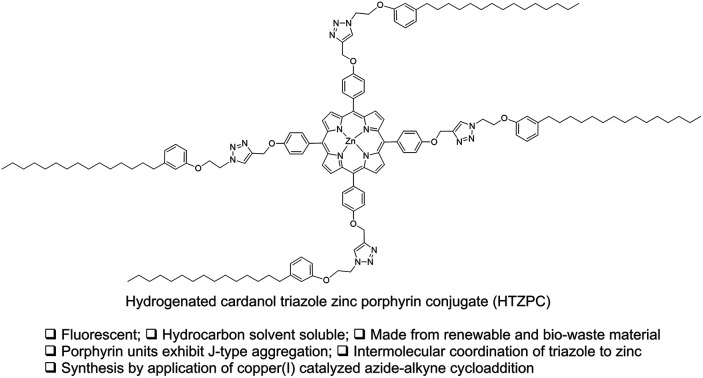

## Introduction

**Fig. 1:**
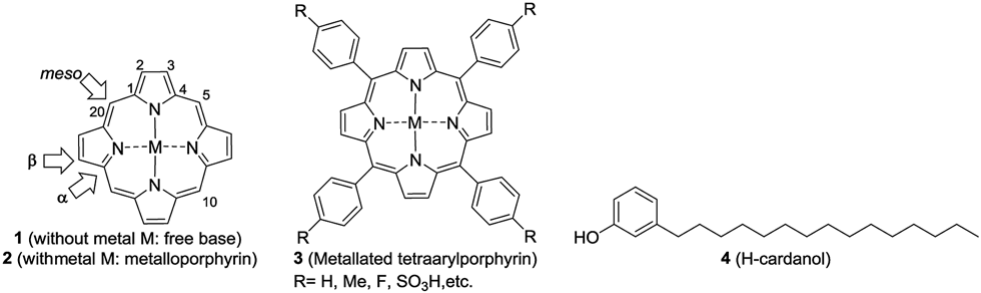
Structures of the porphyrin free base 1 (selected numbering and three types of carbons in the porphyrin ring are highlighted), the metallated porphyrin 2, the metallated tetraphenylporphyrin (TPP) 3 and hydrogenated cardanol (H-cardanol) 4.

Porphyrins constitute an important class of organic entities.^1^ They are colored and therefore are aptly named as pigments of life (porphyria comes from the Greek word for purple).^2^ Since the early years of porphyrin discovery, enormous knowledge has been accumulated to the extent that porphyrins have found applications in various fields like biology, medicine, physics and engineering.^3–5^ Porphyrin ring 1 (Fig. 1) consists of twenty carbon and four nitrogen atoms incorporated into a macrocycle with eleven conjugated double bonds. The insertion of a metal into porphyrin ring 1 leads to the metalloporphyrin 2. Metal insertion leads to drastic changes in the physico-chemical properties of the porphyrin, which often get reflected in its biological properties. Nature is rich in both porphyrins and metallated porphyrins.^6^ For example, the metalloporphyrin ring present in life-essential molecules like heme and chlorophyll plays a quintessential role in their biological processes. Taking advantage of their stability and emission properties, in recent years, a vast number of supra-molecular organic molecules with metalloporphyrin cores have been synthesized in pursuit of their technological application.^7^

Among the porphyrins, the *meso*-aryl substituted porphyrins (3, Fig. 1) form a distinct subgroup.^8^ They are easy to prepare from simple fine chemicals like pyrrole and aryl aldehydes; therefore, they have become extremely popular for model studies of natural porphyrins.

Owing to extended conjugation and symmetry, metalloporphyrin 2 exhibits high-intensity optical absorption in the UV-visible region.^9^ The unique feature of their UV-vis spectra is the occurrence of two distinct bands: one intense band (400 nm, *ε* = >10^5^, Soret band) in the near UV region and a set of two weak bands in the visible region (550–650 nm, *ε* ≤ 10^3^, Q-bands). Apart from their characteristic UV-vis absorption, many metalloporphyrins of type 2 exhibit intense emission spectra and such emission is critically dependent on fine structural changes and the micro-environment. Being disk-like, porphyrin rings tend to stack one above the other in a one-dimensional matrix, which is generally referred to as H-type (like coins in a pillar) and J-type (edge to edge) aggregation.^10,11^ Both the porphyrin rings and central metal ions play a crucial role in the aggregation behavior. The aggregated states of porphyrins exhibit different optical properties (for example, the H-type exhibits a hypsochromic shift and the J-type exhibits a bathochromic shift in the Soret bands) when compared to monomeric species^12,13^

One of the major drawbacks of metalloporphyrins of type 2 is their poor solubility in non-polar solvents, like hexane, or in fat tissue due to the polar nature of the ring system. Since porphyrins of type 1 and metalloporphyrins of type 2 are strongly UV absorbing and fluorescent, one can envisage several applications of fat-soluble porphyrins, *e.g.* in the photodynamic therapy of fat tissue. One way to circumvent the problem is to attach non-polar groups to tetraarylporphyrins at innocuous positions. Indeed, nature has enhanced the fat solubility of chlorophyll by attaching a unit of phytol, a sesquiterpenoid having a 15-C hydrocarbon chain. Even though synthetic porphyrins have been known for decades, strangely there are only very few reports on the synthesis, characterization and evaluation of the optoelectronic properties of hydrocarbon solvent soluble porphyrins.^14–16^

**Fig. 2:**
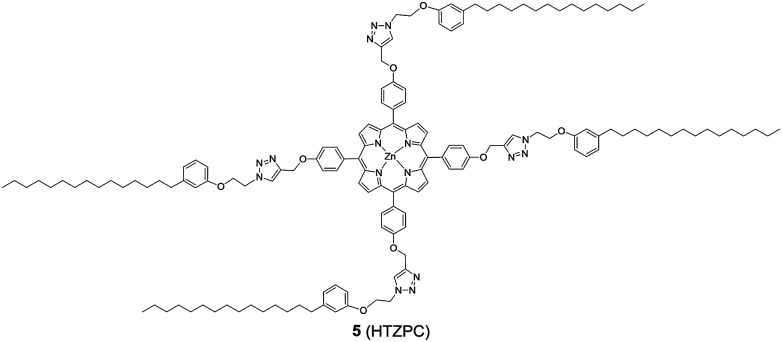
The structure of the H-cardanol-triazole-porphyrin conjugate (HTZPC) 5.

Reflecting our interest in the utilization of hydrogenated cardanol (H-cardanol, 3-pentadecylphenol, 4; Fig. 1) for the synthesis of heterocyclic molecules with hydrophobic properties,^17–19^ we have designed the fat-soluble H-cardanol zinc porphyrin triazole conjugate (HTZPC) 5 (Fig. 2) through linking four units of H-cardanol 4 to zinc tetraphenylporphyrin (3, M = Zn) *via* the triazole units. H-cardanol 4 is a renewable fine chemical derived as a waste by-product of the cashew industry.^20–23^ It can be generated in large quantities *via* two easy steps from cashew nut shell liquid (CNSL), which is derived from the discarded and caustic shells of cashew nuts.^24^ Previously, Sandrino and coworkers have prepared porphyrin-H-cardanol conjugates with the aim of studying self-aggregation in Langmuir films.^25^ However, their conjugates did not have a metal atom in the porphyrin ring or a triazole linker to connect porphyrin with H-cardanol. Attanasi and coworkers prepared zinc porphyrin-H-cardanol conjugates, but their molecules lacked triazole units such as those in 5.^26^ Moreover, they did not study the absorption, emission and solubility characteristics in detail. We reasoned that, as a unit with a lone pair of electrons, triazole could help in the aggregation of porphyrin units *via* inter-molecular coordination with the metal atom in the ring.

**Scheme 1:**
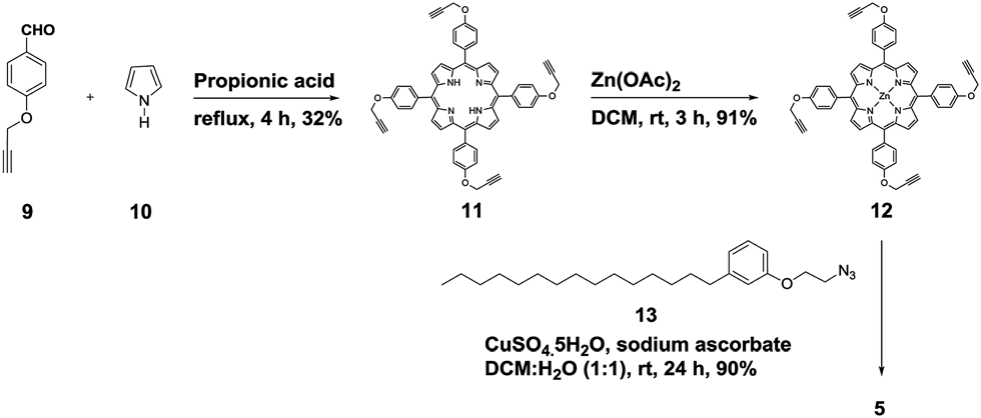
The synthesis of the H-cardanol triazole porphyrin conjugate (HTZPC) 5.

Attanasi's method for the construction of porphyrin rings was used with H-cardanol linked benzaldehyde and pyrrole; the yield from this, however, was low. The metal was introduced in the final step, which we found very difficult to replicate in our system. To circumvent difficulties in the early stages of the construction of the porphyrin ring with H-cardanol units and the late stage introduction of the metal, we planned the early stage construction of zinc-tetraarylporphyrin and the late stage linking of four units of H-cardanol 4*via* triazole tethers through utilizing a copper mediated azide–alkyne cycloaddition reaction (CuAAC; Scheme 1). CuAAC is an archetypical click reaction that provides a convenient and available handle to link two units through regio-chemically well-defined 1,4-disubstituted 1,2,3-triazole units, which are a robust acid–base stable aromatic linker.^27,28^ Moreover, triazole can coordinate to the metal in a porphyrin through the lone pair of electrons on nitrogen, thus altering the optical properties as well as the aggregation behaviour of the resulting conjugate. We chose to prepare zinc(ii) incorporated HTZPC 5 since zinc-porphyrins exhibit intense fluorescence emission.^29^

**Fig. 3:**
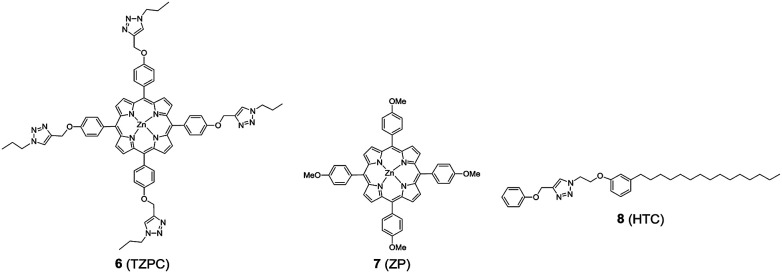
The structures of the triazole-zinc porphyrin conjugate (TZPC) 6, zinc tetra(4-methoxyphenyl)porphyrin (ZP) 7, and the H-cardanol-triazole conjugate (HTC) 8.

In addition to HTZPC 5, we have synthesized the triazole zinc-porphyrin conjugate (TZPC) 6, zinc tetra(4-methoxyphenyl)porphyrin (ZP) 7 ^30^ and the H-cardanol triazole conjugate (HTC) 8 (Fig. 3) to evaluate the contributions of the porphyrin, triazole and H-cardanol units to the absorption, emission and solubility characteristics. We reasoned that while the conjugate HTZPC 5 has all three units, namely H-cardanol, zinc porphyrin and triazole, TZPC 6 has two units (porphyrin and triazole units, but not H-cardanol); ZP 7 has only tetraaryl zinc porphyrin and HTC 8 has two units (H-cardanol and triazole).

## Results and discussion

### Synthesis and characterization of HTZPC **5**, TZPC **6** and HTC **8**

We started the synthesis of HTZPC 5 from the known tetrapropargyloxy porphyrin 12, which was prepared in two steps from 4-propragyloxybenzaldehyde 9 and pyrrole 10 (Scheme 1). The propionic acid mediated condensation of 4-propragyloxybenzaldehyde 9 and pyrrole 10 provided tetraarylporphyrin 11 in moderate (32%) yield. In the next step, we considered the introduction of a Zn^2+^ ion into the porphyrin core of 11 ahead of the CuAAC click reaction with the H-cardanol based azide 13, because the introduction of the zinc ion after the click reaction proved to be unsuccessful. Metalation of the porphyrin propargyl ether 11 with Zn(OAc)_2_·2H_2_O as a zinc(ii) source furnished porphyrin 12 in 91% yield. As anticipated, the UV-vis spectrum of 12 displayed two Q bands at 551 and 589 nm, instead of the four found in the spectrum of 11. The CuAAC reaction of the propargyl ether 12 with the azide 13 ^31^ using CuSO_4_·5H_2_O and sodium ascorbate in a two phase solvent mixture of dichloromethane (DCM) and H_2_O (1 : 1) furnished HTZPC 5 in 90% yield. The conjugate 5 was characterized *via* UV, IR, ^1^H NMR and ^13^C NMR spectra and HRMS analysis.

Principal IR band frequencies (cm^−1^) and assignments for the conjugates are given in Table 1. There were no peaks at around 3500 cm^−1^ and 1000 cm^−1^, as were seen for the porphyrins, which shows that there are no N–H stretching and bending vibrations from the porphyrin core because the hydrogen atoms have been replaced by a Zn metal ion to form Zn–N bonds. Noteworthy frequencies (cm^−1^) due to C–C stretching (benzol, 1630 cm^−1^), the C–H bending of pyrrole (1460 cm^−1^), the –C=N stretching of pyrrole (1350 cm^−1^), the C–O–C bending of alkoxy groups (1240 cm^−1^) and the N=N stretching of triazole (1455 cm^−1^) supported the assigned structure.

**Table 1:** Selected infrared spectral data for HTZPC 5, TZPC 6, ZP 7 and HTC 8. All values are given in cm^−1^

Conjugate	–N=N str (triazole)	C–C str (benzol)	C–H bend (pyrrole)	–C=N str (pyrrole)	C–O–C bend (alkoxy)
HTZPC 5	1628, 1455	1618	1493	1346	1240
TZPC 6	1631, 1490	1601	1460	1351	1215
ZP 7	—	1619	1461	1352	1221
HTC 8	1620, 1458	1590	1492	1351	1245

The ^1^H NMR spectrum of HTZPC 5 in CDCl_3_ displays broad peaks reflecting its macromolecular (MF = C_148_H_192_N_16_O_8_Zn and MW = 2388.65) nature and aggregation. Although broad, the signals due to porphyrin (8 × Cβ-H) and triazole (4H), along with methylene hydrogens from the propargyl ether portion (8H) and two methylenes from azide (2 × 8H), could be deciphered from the spectrum. The ^13^C NMR spectrum of 5 displays a single set of signals from the H-cardanol and triazole units, reflecting the C4 symmetric nature of the molecule.

The synthesis and characterization of TZPC 6 and HTC 8 followed the method described for HTZPC 5, and the details are given in the experimental section.

### The absorption and emission properties and aggregation behaviour of HTZPC 5

HTZPC 5 was synthesized with the intention to evaluate its aggregation in non-polar and polar solvents. In addition, we desired to evaluate its solubility in non-polar solvents. Aggregation in non-polar solvents (*e.g.* hexane) could arise from the π–π stacking of porphyrin units and it could arise in polar solvents (*e.g.* MeOH) due to hydrophobic van der Waals attractive forces between H-cardanol groups. In addition, there is also the possibility of the intra- or intermolecular axial complexation of the triazole unit with the central zinc ion. To evaluate these possibilities, we have recorded and analyzed absorption (UV-vis) and emission (fluorescence) spectra and compared the data with those from TZPC 6, ZP 7 and HTC 8.

**Fig. 4:**
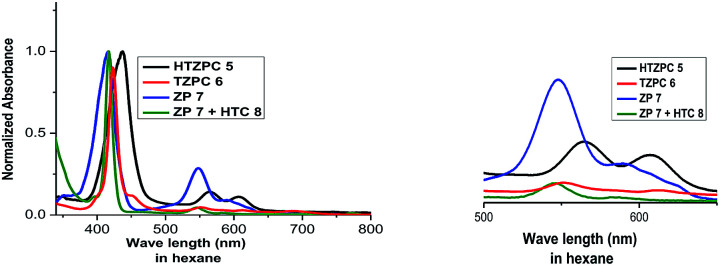
UV-vis spectra of HTZPC 5 (black), TZPC 6 (red), ZP 7 (blue), and ZP 7 + HTC 8 (olive) in hexane at room temperature.

**Fig. 5:**
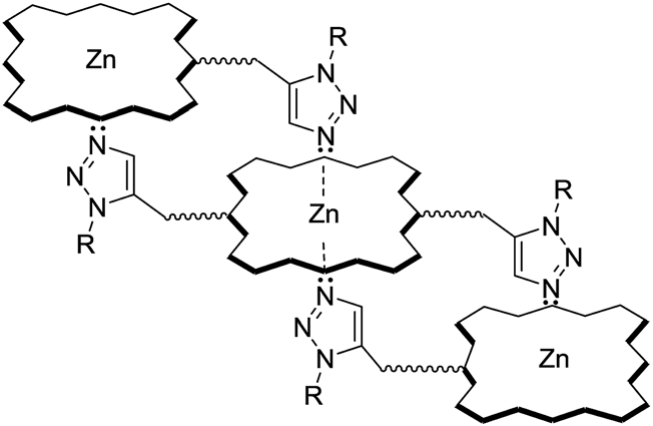
An ensemble of multiple porphyrin units in HTZPC 5.

We recorded UV-vis spectra (Fig. 4) from dilute (4.19 × 10^−4^ M) solutions of HTZPC 5, TZPC 6 and ZP 7 and a 1 : 1 mixture of ZP 7 and HTC 8 in hexane and gathered the data in Table 2. As anticipated for zinc porphyrins, characteristic B (Soret) and Q bands for HTZPC 5, TZPC 6 and ZP 7 occurred in the near-ultraviolet (around 400 nm) and visible (550–620 nm) ranges. They were assigned as resulting from transitions from the ground state (S_0_) to the second lowest excited singlet state (S_2_) for the Soret bands and the ground state (S_0_) to the lowest excited state (S_1_) for the Q bands of the porphyrin chromophores. Conjugates 5 and 6, having porphyrin and triazole units, exhibited similar absorption spectra but these were very different from the absorption spectrum of the parent zinc porphyrin 7, which lacks triazole units. For 5 and 6, the Soret band appeared at around 440 nm, whereas for 7 it appeared at 417 nm. The Q_1_ and Q_2_ bands of 5 and 6 also displayed bathochromic shifts (570 and 610 nm) compared to those of 7 (550 and 590 nm). The bathochromic shift indicates that the Zn ions in 5 and 6 are present as a complex with triazole units. To evaluate the effects of the triazole unit on the UV-vis absorption, we recorded the UV spectrum from an equimolar mixture of ZP 7 and HTC 8 to determine if there was intermolecular influence from the triazole unit in 8 on the porphyrin of 7. The UV spectrum, however, did not show any such effects, as the spectra of 7 and 7 + 8 were almost identical, indicating a lack of intermolecular complexation by the triazole unit. However, for 5 and 6, in which triazole and porphyrin were integral parts of the molecules, there is a possibility for multiple units to form an ensemble generated *via* the coordination of triazole, as shown in Fig. 5. Such an ensemble could grow in three dimensions. We believe that the alternative intra-molecular triazole coordination is not possible, as the tether connecting the triazole unit to the porphyrin in 5 is too short and rigid to bend to a position where the triazole unit is perpendicularly above the zinc ion. Since H-cardanol units can aggregate with themselves through van der Waals attractive forces, the ensemble of 5 shown in Fig. 5 has the higher possibility. This is reflected in the hyperchromic effect seen in 5 compared to 6, as shown in Fig. 4.^32^

**Table 2:** UV-vis spectral data of HTZPC 5, TZPC 6, ZP 7 and ZP 7 + HTC 8 in hexane

Compound	*λ*_max_ [nm] (*ε* in L mol^−1^ cm^−1^)		
	Soret band	Q_1_ band	Q_2_ band
HTZPC 5	437 (151 250)	567 (4228)	607 (3368)
TZPC 6	423 (94 587)	551 (13 889)	612 (12 197)
ZP 7	417 (41 350)	548 (9235)	589 (7324)
ZP 7 + HTC 8	417 (12 746)	545 (6726)	584 (1375)

**Fig. 6:**
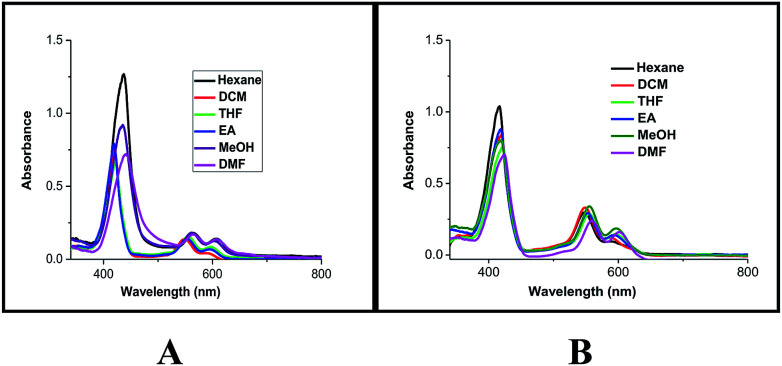
Absorption spectra of (A) HTZPC 5 (4.19 × 10^−4^ M), and (B) ZP 7 (1.25 × 10^−3^ M) in hexane (black), DCM (red), THF (green), EtOAc (blue), MeOH (purple) and DMF (magenta).

To probe the hypothesis for the formation of the intermolecular ensemble shown in Fig. 5, we conducted solvatochromic studies on HTZPC 5 (Fig. 6 and Table 3). Absorption spectra of HTZPC 5 in solvents of different polarity, like hexane (non-polar), dichloromethane (DCM, moderately polar aprotic), tetrahydrofuran (THF, moderately polar aprotic), ethyl acetate (EtOAc; moderately polar aprotic), methanol (MeOH; polar protic) and dimethylformamide (DMF, highly polar aprotic), were recorded. Absorption spectra of the parent ZP 7 in these solvents were used as reference.

**Table 3:** Absorption maxima (wavelength in nm) of the Soret bands of HTZPC 5, TZPC 6, ZP 7, and ZP 7 + HTC 8 (1 : 1) in different solvents

Conjugate	*λ*_max_ a					
	Hexane	DCM	THF	EtOAc	MeOH	DMF
HTZPC 5	437 (438)	420 (417)	421 (418)	419 (415)	435 (414)	441 (444)
TZPC 6	444 (442)	424 (424)	425 (425)	424 (424)	424 (423)	427 (427)
ZP 7	417 (411)	420 (415)	421 (418)	419 (415)	419 (415)	424 (419)
ZP 7 + HTC 8 (1 : 1)	417 (417)	420 (421)	424 (424)	421 (422)	421 (422)	426 (426)

a *λ*_max_ after two days is given in parentheses.

Compared to ZP 7, HTZPC 5 exhibited bathochromic shifts of 20 nm in hexane, 15 nm in MeOH and 17 nm in DMF, but no such shift was noticed in moderately polar solvents like DCM, THF, and EtOAc. The data indicate the aggregation of HTZPC 5 in MeOH (polar protic), DMF (polar aprotic) and hexane (non-polar) solvents. Aggregation in polar solvents (MeOH and DMF) is due to the hydrophobic and van der Waals attractive forces exhibited by H-cardanol. Such aggregation promotes the coordination of triazole units to the Zn ion. In non-polar solvents (hexane), the aggregation is likely to be due to the π–π stacking of porphyrin rings. UV spectra of equimolar mixtures of ZP 7 and HTC 8 were recorded in all six solvents to find out if there was an associative interaction between these two units with a change in the nature of the solvent, but the spectra did not reveal any shifts. The zinc porphyrin 7, as anticipated for metalloporphyrins,^33^ displayed only moderate effects upon changing the solvent polarity. The bathochromic shift of the Soret band of 5 by 7 nm when the solvent was changed from hexane to DMF was in agreement with the similar shift seen for the parent 7.

Absorption spectra of HTZPC 5 and ZP 7 were recorded after two days to observe the nature of the bands as a consequence of possible further aggregation over time. There were no further shifts in the *λ*_max_ values of the Soret and Q bands of 5, but a hypochromic effect was observed (Table 4). The reduction in the intensity of 5 in MeOH was drastic; the absorbance came down from about 1.10 × 10^5^ to 0.09 × 10^5^ L mol^−1^ cm^−1^ due to the precipitation of the conjugate.

**Table 4:** The molar absorption coefficients of HTZPC 5, TZPC 6, ZP 7 and ZP 7 + HTC 8 (1 : 1) in different solvents soon after the preparation of the solutions and after two days of storage at room temperature

Conjugate	*ε* a in L mol^−1^ cm^−1^					
	Hexane	DCM	THF	EtOAc	MeOH	DMF
HTZPC 5	151 250 (84 200)	90 300 (58 400)	79 300 (48 800)	94 350 (62 150)	109 750 (8850)	85 750 (60 350)
TZPC 6	32 154 (23 768)	38 678 (30 245)	73 557 (75 189)	29 314 (34 852)	85 753 (83 951)	93 625 (98 915)
ZP 7	41 350 (9600)	33 400 (22 500)	30 050 (19 050)	35 050 (23 650)	31 950 (18 000)	28 050 (12 700)
ZP 7 + HTC 8 (1 : 1)	127 450 (37 200)	120 100 (109 950)	98 550 (100 300)	126 750 (123 250)	127 500 (121 200)	125 250 (122 600)

a *ε* in L mol^−1^ cm^−1^ after two days is given in parentheses.

**Fig. 7:**
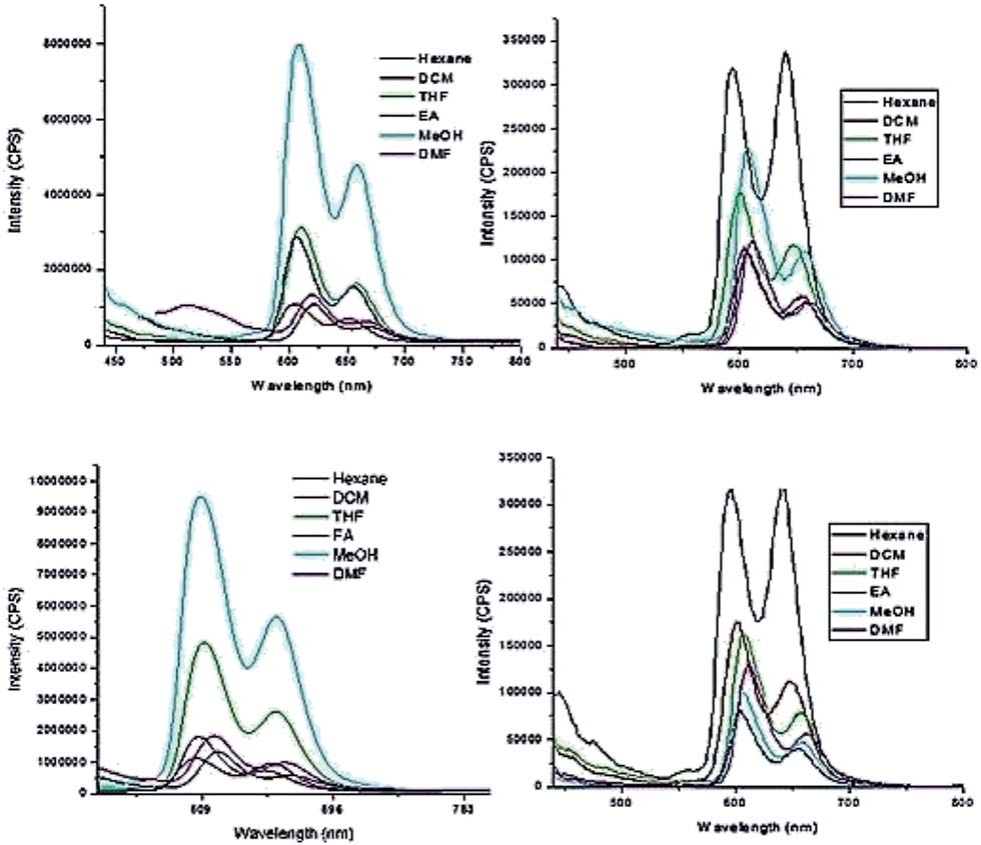
Emission spectra (excited at 422 nm) of HTZPC 5 (left, 4.19 × 10^−4^ M) and ZP 7 (right, 1.25 × 10^−3^ M) soon after the preparation of the solutions (top row) and after two days (bottom row) in hexane (black), DCM (red), THF (green), EtOAc (blue), MeOH (cyan) and DMF (pink).

The fluorescence emission properties of porphyrins are extremely important for their application as sensors. Fluorescent emission is susceptible to drastic changes following minor changes in the structure or environment. Zinc porphyrins in general display intense emission and this property has been used for the design of sensors.^34^ We recorded the emission spectra of HTZPC 5 and ZP 7 (1 × 10^−5^ M) in six solvents, namely hexane, dichloromethane (DCM), tetrahydrofuran (THF), ethyl acetate (EtOAc), methanol (MeOH), and *N*,*N*-dimethylformamide (DMF), which were also employed for recording absorption spectra (Fig. 7 and Table 5). The spectra in all six solvents show two peaks, one strong peak at 607 nm and a weaker one at 658 nm. The former corresponds to a Q(0–0) transition and the latter to a Q(0–1) transition.^35^ The emission intensities of both the bands of HTZPC 5 were at their maximum in methanol. The emission intensities of the bands of the reference 7, on the other hand, were at their maximum in hexane. This observation indicates that in MeOH, 5 undergoes aggregation due to hydrophobic attractive interactions between H-cardanol units and the molecule, as such, becomes more rigid, similar to the example of saturated long chain hydrocarbons. There were bathochromic shifts of about 12 nm for both the Q bands (0–0 and 0–1) of 5 in methanol compared to hexane, which indicates that the excited state of the chromophore is polar and the polar solvent MeOH stabilizes it.

**Table 5:** Emission maximum values (wavelength in nm) of HTZPC 5 and an equimolar mixture of HTC 8 + ZP 7 in different solvents

Entry	*λ*_max_ a					
	Hexane	DCM	THF	EtOAc	MeOH	DMF
HTZPC 5	607, 658 (619, 667)	605, 650 (606, 651)	610, 658 (610, 658)	607, 656 (607, 656)	608, 658 (608, 658)	619, 664 (617, 664)
HTC 8 + ZP 7	491 (496)	606, 656 (606, 657)	608, 658 (611, 658)	606, 657 (605, 656)	608, 658 (610, 658)	613, 663 (615, 662)

a *λ*_max_ after two days is given in parentheses.

**Fig. 8:**
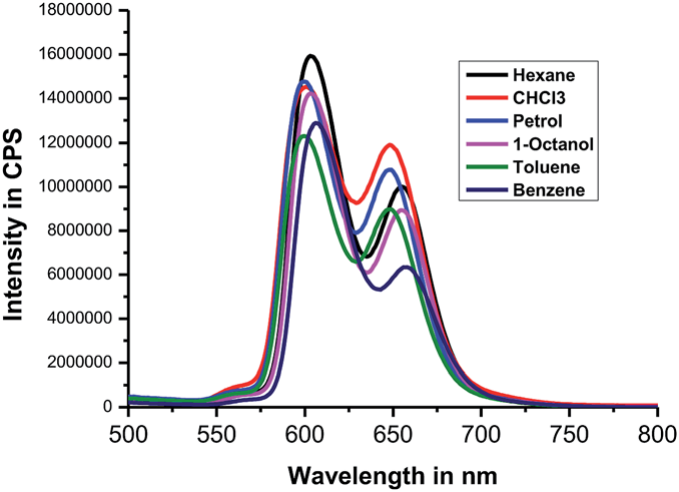
Emission spectra (excited at 422 nm) of HTZPC 5 (4.19 × 10^−4^ M) in the non-polar solvents hexane (black), CHCl_3_ (red), petrol (blue), 1-octanol (magenta), toluene (olive blue) and benzene (navy green).

Next, we recorded fluorescence emission spectra of HTZPC 5 in six non-polar solvents, namely hexane, petrol, benzene, toluene, chloroform and 1-octanol. We reasoned that the fluorescence emission properties of HTZPC 5 in petrol could be used to detect impurities and tests in 1-octanol could indicate their use as a probe in fatty tissue. Although deep studies were not conducted, our readings (Fig. 8, Table 6) indicate that the emission of HTZPC 5 in all six solvents is similar. Emission in 1-octanol is similar to that in hexane rather than methanol. In benzene and toluene, the intensity of the Q(0–1) transition located at around 650 nm is lower than in hexane or petrol.

**Table 6:** Emission maximum values (wavelength in nm) of HTZPC 5 in non-polar solvents

S. no	Solvent	*λ*_max_ (nm)	Intensity in CPS
1	Hexane	603	1.5 × 10^7^
		655	1.0 × 10^7^
2	Petrol	600	1.4 × 10^7^
		648	1.0 × 10^7^
3	Benzene	606	1.2 × 10^7^
		657	6.3 × 10^6^
4	Toluene	600	1.2 × 10^7^
		646	8.0 × 10^6^
5	CHCl_3_	600	1.4 × 10^7^
		648	1.0 × 10^7^
6	1-Octanol	603	1.4 × 10^7^
		655	8.0 × 10^6^

### The solubility characteristics of HTZPC **5** in different solvents

**Fig. 9:**
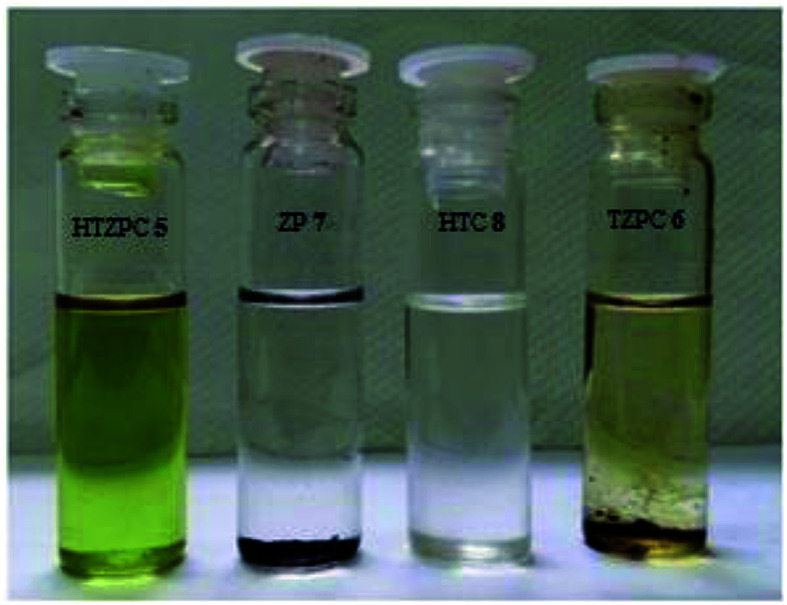
Photographs of 5 mg each of HTZPC 5, TZPC 6, ZP 7 and HTC 8 in hexane. While HTZPC 5 and HTC 8 were freely soluble, TZPC 6 and ZP 7 were practically insoluble.

**Fig. 10:**
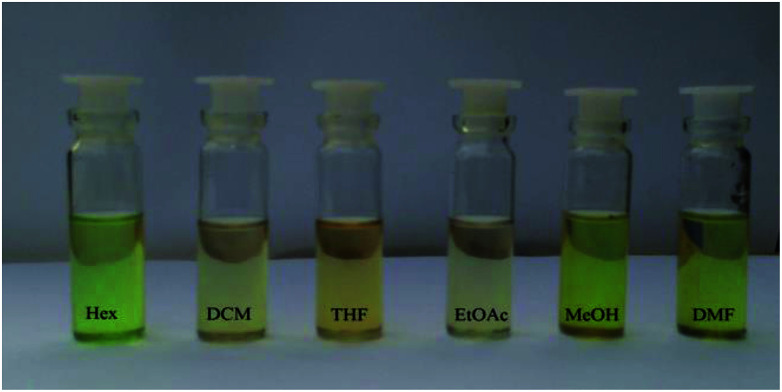
Photographs of HTZPC 5 dissolved in six different solvents, namely hexane (Hex), dichloromethane (DCM), tetrahydrofuran (THF), ethyl acetate (EtOAc), methanol (MeOH) and dimethylformamide (DMF).

Next, we studied the solubility of HTZPC 5 in the six solvents employed for recording absorption spectra (*vide supra*). We found that 5 mg each of HTZPC 5 and HTC 8 were freely and completely soluble in 3 mL of hexane (Fig. 9). On the other hand, TZPC 6 and ZP 7, which do not have H-cardanol units, were practically insoluble. In moderately polar solvents, like DCM, THF and EtOAc, 5 was freely soluble. As anticipated for a fat-like molecule, the solubility of 5 in polar solvents like MeOH and DMF was low (about 0.5 mg in 3 mL). Interestingly, dilute solutions of 5 in hexane, MeOH and DMF were green in color, whereas solutions in DCM, THF and EtOAc were purple (Fig. 10). The change in color in hexane, MeOH and DMF can be attributed to the perturbation of the chromophore in 5 by triazole units as a consequence of aggregation.

## Conclusions

In conclusion, we have successfully synthesized the H-cardanol triazole porphyrin conjugate HTZPC 5. The macromolecule was characterized *via* spectroscopic and analytical methods. The optoelectronic characteristics were evaluated using UV-vis and fluorescence emission spectra recorded in various solvents. A comparison of the results with data collected from the sibling molecules triazole-zinc porphyrin conjugate TZPC 6, tetra-C(4)-methoxy TPP (ZP) 7, and H-cardanol-triazole conjugate HTC 8 showed that there was J-type aggregation in non-polar solvents and highly polar solvents. The aggregation is induced by van der Waals attractive forces between H-cardanol units in polar solvents and π–π stacking interactions between porphyrin units in non-polar solvents. An incisive analysis of the absorption and emission spectra revealed that triazole units could stabilize the zinc porphyrin through intermolecular complexation. Solubility studies revealed that the H-cardanol units make porphyrin 5 soluble in hexane. Since HTZPC 5 is easy to synthesize, is fat soluble and shows good fluorescence emission, we anticipate that it will be used for applications in the medical field that could include the photodynamic therapy of fat tissue.^36,37^

## Experimental section

### Materials and physical measurements

All reagents and solvents were purchased from Sigma-Aldrich, E-Merck or SRL, India. Melting points were uncorrected and were determined using open-ended capillary tubes with a VEEGO VMP-DS instrument. TLC was performed with silica gel G (SRL, India) or silica gel GF-254 (E-Merck) using a solution of ethyl acetate in hexane as the eluent. The spots were visualized through short exposure to iodine vapor or UV light. Column chromatography was carried out on silica gel (100–200 mesh, SRL Chemicals, India) using an increasing percentage of ethyl acetate in hexane. UV spectra were recorded as dilute solutions in different solvents (hexane (Hex), dichloromethane (DCM), tetrahydrofuran (THF), ethyl acetate (EtOAc), methanol (MeOH) and dimethylformamide (DMF)) using a Shimadzu UV-2450 double beam spectrometer. Infrared (IR) spectra were recorded in KBr pellet form using a Bomem MB104 spectrometer or Nicolet-6700 spectrometer. ^1^H NMR (400 MHz), ^13^C NMR (100 MHz) and DEPT spectra were recorded in CDCl_3_, CCl_4_ : CDCl_3_ (1 : 1) or DMSO-D_6_ using a Bruker 400 MHz NMR spectrometer with TMS (0 ppm)/CHCl_3_/CDCl_3_ as the internal standard; *J* values are in Hz. ^1^H NMR data is reported as follows: chemical shift, multiplicity (s = singlet, d = doublet, t = triplet, q = quartet and m = multiplet), coupling constant and integration. High-resolution mass spectra were recorded using an Agilent Q-TOF Micro mass spectrometer in electro-spray ionization mode. Cardanol oil was purchased from Sabari Industries Sedurapet, Pondicherry. Cardanol was hydrogenated using Parr hydrogenation apparatus at the Indian Institute of Technology Madras, Chennai to obtain H-cardanol.^38^ The H-cardanol azide was prepared from H-cardanol *via* a two-step synthesis. The TPP derivatives 5,10,15,20-tetra(4-propargyloxy-phenyl)porphyrin 11 and 5,10,15,20-tetra(4-propargyloxy-phenyl)porphyrin·Zn 12 were synthesized following literature procedures.^39^

#### General procedure for the CuAAC reaction

##### The synthesis of the hydrogenated cardanol triazole zinc porphyrin conjugate (HTZPC) 5


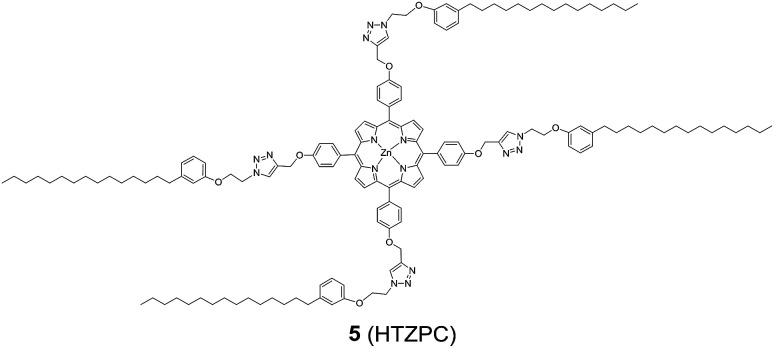
To the Zn TPP derivative 12 (50 mg, 0.06 mmol) in 5 mL of DCM/water (1 : 1), H-cardanol azide 13 (127 mg, 0.28 mmol), CuSO_4_·5H_2_O (2 mg, 0.006 mmol) and sodium ascorbate (3 mg, 0.012 mmol) were added, and the reaction mixture was stirred at rt for three days (72 h). After the completion of the reaction, the reaction mixture was extracted with DCM (20 mL). The DCM solution was separated, washed with brine and dried over Na_2_SO_4_. The removal of DCM under reduced pressure provided the crude dark waxy product. Purification through column chromatography using CHCl_3_/CH_3_OH (99 : 1) as the eluent furnished HTZPC 5 (119 mg, 90%) as a purple colored solid: mp: 187 °C; *R*_f_ = 0.6 (95% DCM/MeOH); IR (KBr) *ν*_max_: 2923, 2852, 1628, 1493, 1240, 994 cm^−1^; ^1^H NMR (400 MHz, CDCl_3_) *δ*: 8.91 (s, 2H), 8.08 (d, *J* = 7.6 Hz, 2H), 7.54 (s, 1H), 7.16–7.14 (m, 3H), 6.80 (d, *J* = 7.2 Hz, 1H), 6.66–6.60 (m, 2H), 4.56 (s, 2H), 4.43 (s, 2H), 4.12 (s, 2H), 2.55–2.53 (m, 2H), 1.57 (br, s, 2H), 1.27–1.22 (m, 24H), 0.87 (br, t, 3H) ppm; ^13^C NMR (100 MHz, CDCl_3_) *δ*: 157.8, 157.7, 150.5, 145.1, 134.7, 136.5, 131.7, 129.5, 123.4, 122.0, 120.3, 114.7, 112.8, 111.6, 66.0, 61.6, 49.8, 36.1, 32.0, 31.5, 29.8, 29.77, 29.73, 29.6, 29.5, 29.4, 22.8, 14.2 ppm; HRMS (ESI): calcd for C_148_H_192_N_16_O_8_Zn [M + H]: 2388.6384 amu, found: 2388.4509 amu.

##### The synthesis of the triazole zinc porphyrin conjugate (TZPC) 6


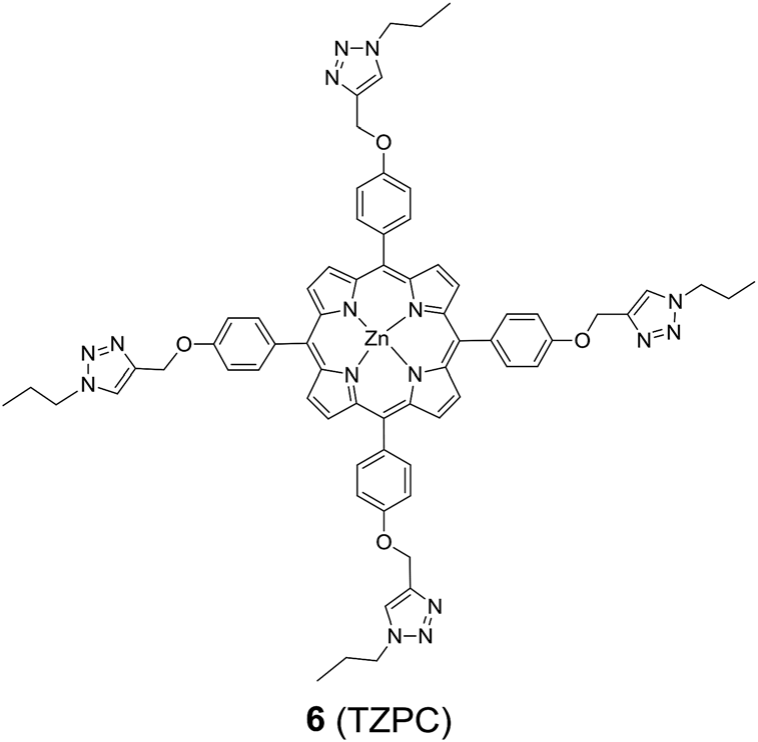
To the Zn TPP derivative 12 (15 mg, 0.016 mmol) in 3 mL of a two-phase mixture of DCM and water (1 : 1), propyl azide (8.1 mg, 0.096 mmol), CuSO_4_·5H_2_O (2 mg, 0.006 mmol) and sodium ascorbate (4 mg, 0.012 mmol) were added, and the resulting reaction mixture was stirred at rt for three days until the completion of the reaction (TLC). Work-up and purification through column chromatography according to the general procedure described above was conducted, and TZPC 6 was obtained (16 mg, 80%) as a purple colored solid: mp: 198 °C; *R*_f_ = 0.4 (95% DCM/MeOH); IR (KBr) *ν*_max_: 2964, 2930, 2874, 1604, 1505, 1462, 1261, 1233, 1175, 993, 803 cm^−1^; ^1^H NMR (400 MHz, DMSO-D_6_) *δ*: 8.79 (s, 2H), 8.36 (s, 1H), 8.05 (d, *J* = 8.0 Hz, 2H), 7.42 (d, *J* = 8.0 Hz, 2H), 5.41 (s, 2H), 4.43 (t, *J* = 8.0 Hz, 2H), 1.95 (q, *J* = 8.0 Hz, 2H), 0.95 (t, *J* = 8.0 Hz, 3H) ppm; ^13^C NMR (100 MHz, DMSO-D_6_) *δ*: 157.5, 149.4, 142.6, 135.3, 135.0, 131.2, 124.4, 119.7, 112.7, 61.4, 51.0, 23.2, 10.8 ppm; HRMS (ESI): calcd for C_68_H_64_N_16_O_4_Zn [M + H]: 1232.46 amu, found: 1233.4664 amu.

##### The synthesis of 1-(2-(3-pentadecylphenoxy)ethyl)-4-(phenoxymethyl)-1*H*-1,2,3-triazole (HTC) 8


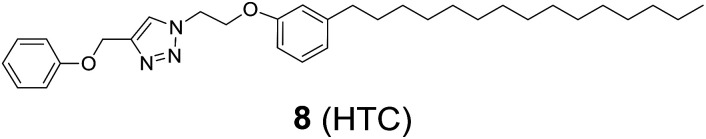
The CuAAC reaction of phenoxy propargyl ether (0.1 g, 0.292 mmol) with H-cardanol azide 13 (0.157 g, 0.350 mmol) in the presence of CuSO_4_·5H_2_O (0.007 g, 0.0292 mmol) and sodium ascorbate (0.010 g, 0.053 mmol) in 5 mL of a two-phase mixture of DCM and water (1 : 1) provided the H-cardanol triazole conjugate (HTC) 8 (0.212 g, 94%) as a colorless paste: *R*_f_ = 0.5 (70% hexane/EtOAc); IR (KBr) *ν*_max_: 3037, 2957, 2919, 2850, 1604, 1582, 1497, 1297, 1250, 1157, 1049, 874, 750 cm^−1^; ^1^H NMR (400 MHz, CDCl_3_) *δ*: 7.78 (s, 1H), 7.28–7.24 (m, 2H), 7.14 (t, *J* = 7.6 Hz, 1H), 6.97–6.92 (q, 3H), 6.78–6.77 (d, 1H), 6.65–6.61 (dd, 2H), 5.20 (s, 2H), 4.75–4.71 (q, 2H), 4.34–4.31 (q, 2H), 2.56–2.52 (t, *J*, = 8 Hz, 2H), 1.59–1.56 (t, *J* = 7.6 Hz, 3H), 1.30–1.25 (m, 25H), 0.89–0.86 (q, 3H) ppm; ^13^C NMR (100 MHz, CDCl_3_) *δ*: 158.4, 157.9, 145.0, 129.6, 129.5, 123.9, 122.1, 121.3, 114.98, 114.93, 111.7, 66.4, 62.1, 50.0, 36.2, 32.1, 31.6, 29.9, 29.8, 29.7, 29.6, 22.9, 14.3 ppm; HRMS (ESI): calcd for C_32_H_47_N_3_O_2_ [M + H]: 506.3747 amu, found: 506.3742 amu.

## Conflicts of interest

There are no conflicts to declare.

## Supplementary Material

RA-009-C8RA09998G-s001
