# Supplementary material for: Synthesis and physico-chemical properties of a H-cardanol triazole zinc porphyrin conjugate
Source: RSC Adv. 2019 Feb 5;9(8):4499–506. doi: 10.1039/c8ra09998g (PMC9060588; doi:10.1039/c8ra09998g)
Supplement: RA-009-C8RA09998G-s001 [file RA-009-C8RA09998G-s001.pdf]

Electronic Supplementary Material (ESI) for RSC Advances.
This journal is © The Royal Society of Chemistry 2019

SUPPORTING INFORMATION

Synthesis and physico-chemical properties of H-cardanol triazole zinc porphyrin conjugate

H. Surya Prakash Rao,* M. Kamalraj, M. Prabakaran

Department of Chemistry, Pondicherry University, Pondicherry – 605 014, India

hspr@yahoo.com

1. Synthetic procedures and analytical data

Phenyl propargyl ether: To the solution of phenol (0.5 g, 5 mmol) in 15 mL of acetone K_2CO_3 (0.63 g, 7.2 mmol, 1.4 equiv) was added at rt. After 15 min, propargyl bromide (614 mg, 10 mmol, 2.0 equiv) was added drop-wise and the mixture was heated under reflux for 3 h. Water (20 mL) was added to cooled reaction mixture and acetone was removed under reduced pressure. The resulting turbid aqueous solution was extracted with dicholoromethane (CH_2Cl_2, 20 mL x 2). Pooled CH_2Cl_2 solution was washed with brine, dried over anhydrous Na_2SO_4, filtered and concentrated under reduced pressure to result in phenyl propargyl ether as a colourless liquid in 91% yield (0.720 g). R_f = 0.5 (hexanes). IR Data (ν): 3292, 3065, 2921, 2123, 1595, 1493, 1215, 1037, 755, 689 cm^{-1}; ^{1}H NMR (400 MHz, $CDCl_3$) δ 7.32 (t, *J* = 8.0 Hz, 2H), 7.00 (t, *J* = 8.9 Hz, 3H), 4.71 (d, *J* = 2.3 Hz, 2H), 2.53 (t, *J* = 2.3 Hz, 1H) ppm; ^{13}C NMR (100 MHz, $CDCl_3$) δ 157.6, 129.6, 121.6, 114.9, 78.7, 75.5, 55.8 ppm.

4-(Propargyloxy)benzaldehyde (9): To the solution of 4-hydroxybenzaldehyde (2.0 g, 16 mmol) in acetone (50 mL) K_2CO_3 (3.1 g, 20 mmol, 1.4 equiv) was added at rt. After 15 min, propargyl bromide (2.5 mL, 30 mmol, 2.0 equiv) was added drop-wise. Resulting reaction mixture was heated under reflux for 4 h. Then the solvent was evaporated in vacuo. The residue water (20 mL) was added and the resulting turbid solution was extracted with EtOAc (2 x 20 mL). The combined organic layers were washed with water and brine, dried over anhydrous Na_2SO_4, filtered and concentrated under reduced pressure. The resulting compound **9** was obtained in 95% yield (1.6 g) as light yellow colour solid. R_f = 0.5 (20% EtOAc in hexanes). Mp: 84-86 °C. IR Data (ν): 3212, 2832, 2808, 2749, 2121, 1682, 1603, 1575, 1252, 1169, 1021, 828, 700, 653 cm^{-1}; ^{1}H NMR (400 MHz, $CDCl_3$) δ 9.88 (s, 1H), 7.84 (d, *J* = 8.9 Hz, 2H), 7.07 (d, *J* = 8.7 Hz, 2H), 4.77 (d, *J* = 2.4 Hz, 2H), 2.57 (s, 1H) ppm; ^{13}C NMR (100 MHz, $CDCl_3$) δ 190.9, 162.45, 131.9, 130.6, 115.2, 77.6, 76.4, 56.0 ppm. HRMS (ESI) Calcd for $C_{10}H_8O_2$ [M+H] 161.0524 amu, found 161.0627 amu.

1-(2-Bromoethoxy)-3-pentadecylbenzene: H-Cardanol (1.0 g, 3.2 mmol) was dissolved in dibromoethane (3 mL, 42 mmol, 13.0 equiv) by heating to 70 °C for 30 min. To the resulting solution KOH (0.213 g, 4 mmol, 1.0 equiv) was added and temperature was maintained at 70 °C for 14 h. Then excess dibromoethane was evaporated in vacuo. The residue was extracted with CH_2Cl_2 and washed with water and brine, dried over anhydrous Na_2SO_4, filtered and concentrated under reduced pressure. The resulting H-cardanol bromide was obtained as white colour solid in 91% yield (1.19 g). R_f = 0.5 (hexanes); Mp: 58-60 °C. IR Data (ν): 2925, 2853, 1604, 1587, 1449, 1257, 1157, 1026, 775, 694 cm^{-1}; ^{1}H NMR (400 MHz, $CDCl_3$) δ 7.20 (t, *J* = 7.8 Hz, 1H), 6.82 (d, *J* = 7.6 Hz, 1H), 6.74 (dd, *J* = 3.4 Hz, 2H), 4.18 – 4.11 (m, 2H), 3.59 (t, *J* = 5.0 Hz, 2H), 2.68 – 2.45 (m, 2H), 1.27 (s, 26H), 0.89 (t, *J* = 6.8 Hz, 3H) ppm; ^{13}C NMR (100 MHz, $CDCl_3$) δ 158.3, 144.9, 129.3, 121.6, 115.0, 111.5, 66.9, 36.3,

32.0, 31.5, 29.84, 29.8, 29.7, 29.6, 29.5, 29.4, 29.3, 22.8, 14.2 ppm. HRMS (ESI) Calcd for $C_{23}H_{39}BrO$ [M+H] 410.2184 amu, found 411.2184 amu.

H-Cardanol azide (13): To the solution of H-cardanol bromide (0.5 g, 1.2 mmol) in DMSO (5 mL) 18-Crown-6 (0.032 g, 0.1 equiv), Bu_4NBr (0.039 g, 0.1 equiv), and NaN_3 (0.087 g, 1.3 mmol, 1.1 equiv) were added sequentially. Resulting mixture was heated to 70 °C and stirred overnight. To the cooled reaction mixture was diluted with CH_2Cl_2 (50 mL) and the white precipitate that formed was removed by filtration. The resulting clear solution was washed with brine (3 x 50 mL) .The organic portion was dried over anhydrous Na_2SO_4, filtered and concentrated under reduced pressure to result in **13** as a colourless liquid in 94% yield (0.430 g). R_f = 0.5 (hexanes). IR Data (ν): 3034, 2925, 2853, 2104, 1606, 1586, 1449, 1259, 1158, 1064, 778, 694 cm^{-1}; ^1H NMR (400 MHz, $CDCl_3$) δ 7.20 (t, *J* = 7.8 Hz, 1H), 6.82 (d, *J* = 7.6 Hz, 1H), 6.74 (dd, *J* = 11.6, 3.4 Hz, 2H), 4.18 – 4.11 (m, 2H), 3.59 (t, *J* = 5.0 Hz, 2H), 2.68 – 2.45 (m, 2H), 1.27 (s, 26H), 0.89 (t, *J* = 6.8 Hz, 3H) ppm; ^{13}C NMR (100 MHz, $CDCl_3$) δ 158.3, 144.9, 129.3, 121.6, 115.0, 111.5, 66.9, 50.3, 36.3, 32.0, 31.5, 29.84, 29.82, 29.7, 29.6, 29.5, 29.4, 22.8, 14.2 ppm. HRMS (ESI) Calcd for $C_{23}H_{39}N_3O$ [M+H] 374.3093 amu, found 374.3175 amu.

5,10,15,20-Tetra(4-propargyloxyphenyl)porphyrin (11): Propionic acid (100 mL) taken in 500 mL round-bottomed flask under a blanket of dry N_2 was heated to 120 °C. To the hot solvent, the solution of 4-propargyloxybenzaldehyde **9** (3.6 g, 22.5 mmol) and freshly distilled pyrrole **10** (1.6 mL, 22.5 mmol) in 5 mL of propionic acid was added drop-wise. After 1 h, the mixture was cooled to rt and stored in refrigerator for 6 h. Addition of methanol (50 mL) purple colored solid **11** resulted which was filtered (yield = 0.976 g, 25%). R_f = 0.5 (50% hexanes in CH_2Cl_2). Mp: 202 - 204 °C, IR Data (ν): 3442, 3285, 2927, 2831, 2119, 1628, 1604, 1505, 1472, 1351, 1218, 1026, 801, 642 cm^{-1}; ^1H NMR (400 MHz, $CDCl_3$) δ 8.96 (S, 1H), 8.129 (d ,*J* =8.8Hz, 2H), 7.34 (d, *J* = 8.8Hz, 2H),4.96 (d, *J* = 2.4, 2H), 2.69-2.68(t, *J* = 2.4Hz, 1H) ppm; ^{13}C NMR (100 MHz, $CDCl_3$) δ 157.4, 150.5,136.2,135.5, 132.0, 120.6, 113.1,78.8, 75.9, 56.2 ppm. HRMS (ESI) Calcd for $C_{56}H_{38}N_4O_4$ [M+H] 831.2893 amu, found 831.2978 amu.

5,10,15,20-Tetra(4-propargyloxyphenyl)porphyrin Zn (12): A solution of zinc acetate (0.551 g, 3.01 mmol) in methanol (3 mL) was added drop-wise to a solution of tetra(4-propargyloxyphenyl)porphyrin **11** (0.500 g, 0.60 mmol) in $CHCl_3$ (5 mL) and resulting solution was heated to reflux under a blanket of dry N_2 for 2 h. After completion of reaction (TLC), the reaction mixture was concentrated to remove MeOH and $CHCl_3$. Resulting viscous mass was diluted with CH_2Cl_2 (20 mL) and the resulting solution was washed with water (3 x 20 mL), dried over Na_2SO_4, filtered and concentrated under reduced pressure. The crude mixture was subjected to column chromatography (50% hexanes in CH_2Cl_2) to give **12** as a purple solid in 95% yield (0.495 g). R_f = 0.5 (50%, hexanes in CH_2Cl_2). Mp: 167-168 °C. IR Data (ν): 3281, 2920, 2120, 1628, 1603, 1502, 1447, 1335, 1217, 1024, 802, 642 cm^{-1}; ^1H NMR (400 MHz, $CDCl_3$) δ 8.96 (S, 1H), 8.129 (d ,*J* =8.8Hz, 2H), 7.34 (d, *J* = 8.8Hz, 2H),4.96 (d, *J* = 2.4, 2H), 2.69-2.68 (t, *J* = 2.4Hz, 1H) ppm; ^{13}C NMR (100 MHz, DMSO - d_6) δ 157.4, 150.5,136.2,135.5, 132.0, 120.6, 113.1,78.8, 75.9, 56.2 ppm. HRMS (ESI) Calcd for $C_{56}H_{38}N_4O_4Zn$ [M+H] 893.2028amu, found 893.2107amu.

5,10,15,20-Tetra(4-methoxyphenyl) porphyrin: Propionic acid (100 mL) taken in 500 mL round-bottomed flask under a blanket of dry N_2 was heated to 120 °C. To the hot solvent, the solution of 4-anisaldehyde (3.6 g, 22.5 mmol), freshly distilled pyrrole **10** (1.7 mL, 22.5 mmol) in 5 mL of propionic acid was added drop-wise. After completion of the reaction

(TLC, 2 h) the mixture was cooled to rt and stored in refrigerator for 6 h. Upon addition of methanol (50 mL) purple colored solid resulted which was filtered (yield = 1.24 g, 22%). R_f = 0.5 (50% hexanes in CH_2Cl_2). Mp: 203-205 °C. IR Data (ν): 3442, 3285, 2927, 2831, 2119, 1628, 1604, 1505, 1472, 1351, 1218, 1026, 801, 642 cm^{-1}; ^1H NMR (400 MHz, $CDCl_3$) δ 8.96 (s, 1H), 8.12 (d ,*J* =8.0Hz, 2H), 7.92 (s, 2H),4.1 (s, 3H) ppm; ^{13}C NMR (100 MHz, $CDCl_3$) δ 159.3, 150.6, 135.5, 135.4, 132.0, 120.8, 112.9, 55.7 ppm.

5,10,15,20-Tetra(4-methoxyphenyl) porphyrin Zn (7): A solution of zinc acetate (0.621 g, 3.01 mmol) in methanol (3 mL) was added drop-wise to a solution of tetra(4-methoxyphenyl) porphyrin (0.500 g, 0.60 mmol) in $CHCl_3$ (5 mL). Resulting solution was refluxed under N_2 for 2 h. After completion of reaction, the reaction mixture was concentrated under reduced pressure. Resulting viscous mass was diluted with CH_2Cl_2 (20 mL) and the resulting solution was washed with water (3 x 20 mL), dried over Na_2SO_4, filtered and concentrated under reduced pressure. The crude product was purified by column chromatography (10% MeOH in $CHCl_3$) to give **7** as a purple solid in 95% yield (0.505 g). R_f = 0.5 (10% MeOH in $CHCl_3$). Mp: 167-168 °C. IR Data (ν): 3442, 3285, 2927, 2831, 2119, 1628, 1604, 1505, 1472, 1351, 1218, 1026, 801, 642 cm^{-1}; ^1H NMR (400 MHz, $CDCl_3$) δ 8.96 (s, 1H), 8.12 (d ,*J* =8.0Hz, 2H), 7.92 (s, 2H),4.1 (s, 3H) ppm; ^{13}C NMR (100 MHz, $CDCl_3$) δ 159.3, 150.6,135.5,135.4,132.0, 120.8, 112.9, 55.7 ppm. HRMS (ESI) Calcd for $C_{56}H_{38}N_4O_4Zn$ [M+H] 893.2028 amu, found 893.2107 amu.

H-Cardanol triazole porphyrin conjugate (HTZPC) (5): To Zn metallotetraphenylporphyrin propargyl ether **12** (0.050 mg, 0.06 mmol) taken in 5 mL of mixture of CH_2Cl_2 and water (1:1) cardanol azide **13** (0.127 mg, 0.28 mmol), $CuSO_4.5H_2O$ (0.02 mg, 0.006 mmol) and sodium ascorbate (0.03 mg, 0.012mmol) were added sequentially. Resulting reaction mixture was allowed to stir at rt for three days. After completion of the reaction, the reaction mixture was diluted with CH_2Cl_2 (20 mL). The CH_2Cl_2 layer was washed with brine (10 mL), dried over Na_2SO_4. Evaporation of CH_2Cl_2 resulted in crude product which was purified by column chromatography with 10% MeOH in $CHCl_3$ to furnish Zn pophyrin **5** as purple colored solid in 86% yield (0.115 g). R_f = 0.6 (10% MeOH in $CHCl_3$). Mp: 193-195 °C. IR Data (ν): 2922, 2851, 1628, 1605, 1489, 1462, 1389, 1337, 1244, 993, 800, 693cm^{-1}; ^1H NMR (400 MHz, $CDCl_3$) δ 8.91 (s, 2H), 8.08 (d, *J* = 7.6 Hz, 2H), 7.54 (s, 1H), 7.16-7.14 (m, 3H), 6.80 (d, *J* = 7.2 Hz, 1H), 6.66-6.60 (m, 2H), 4.56 (s, 2H), 4.43 (s, 2H), 4.12 (s, 2H), 2.55-2.53 (m, 2H), 1.57 (br, s, 2H), 1.27-1.22 (m, 24H), 0.87 (br, t, 3H) ppm; ^{13}C NMR (100 MHz, $CDCl_3$) δ 157.8, 157.7, 150.5, 145.1, 143.7 134.7, 136.5, 131.7, 129.5, 123.4, 122.0, 120.3, 114.7, 112.8, 111.6, 66.0, 61.6, 49.8, 36.1, 32.0, 31.5, 29.8, 29.77, 29.73, 29.6, 29.5, 29.4, 22.8, 14.2 ppm. HRMS (ESI) Calcd for $C_{148}H_{192}N_{16}O_8Zn$ [M+H] 2388.6384 amu, found 2388.4509 amu.

Triazole Zinc Porphyrin Conjugate (TZPC) (6): To zinc tetraphenylporphyrin propargyl ether **12** (0.020 g, 0.02 mmol) taken in a mixture of 5 mL of CH_2Cl_2 and water (1:1) propyl azide (0.04 g, 0.3 mmol), $CuSO_4.5H_2O$ (0.02 g, 0.006 mmol) and sodium ascorbate (0.03 g, 0.012mmol) were added sequentially. Resulting mixture allowed to stir at rt for three days. After completion of the reaction, the reaction mixture was diluted with CH_2Cl_2 (20 mL) and the separated CH_2Cl_2 layer was washed with brine (10 mL), dried over Na_2SO_4, and solvent removed under reduced pressure to result in crude product. Upon purification by column chromatography using 10% MeOH in $CHCl_3$ as a eluent TZPC **6** resulted as purple colored solid in 86% yield (0.033 g). R_f = 0.4 (10% MeOH in $CHCl_3$). Mp: 172 °C. IR Data (ν): 2964, 2930, 2874, 1628, 1605, 1462, 1336, 1233, 1175, 993, 803 cm^{-1}; ^1H NMR (400 MHz, $CDCl_3$) δ 8.79 (s, 2H), 8.36 (s, 1H), 8.05 (d, *J* = 8.0 Hz, 1H), 7.43 (d, *J* = 8.0 Hz, 2H), 5.41

(s, 2H), 5.43 (t, J = 8.0 Hz, 2H), 1.95 (q, J = 8.0 Hz, 2H), 0.95 (t, J = 8.0 Hz, 3H) ppm; ^{13}C NMR (100 MHz, $CDCl_3$) δ 157.5, 149.4, 142.6, 135.3, 135.0, 131.2, 124.4, 119.7, 112.7, 61.4, 51.1, 23.2, 10.8 ppm. HRMS (ESI) Calcd for $C_{68}H_{64}N_{16}O_4Zn$ [M+H] 1232.4588 amu, found 1232.4664 amu.

1-(2-(3-Pentadecylphenoxy)ethyl)-4-(phenoxymethyl)-1H-1,2,3-triazole (HTC) (8): To the solution of phenoxy propargyl ether (0.1 g, 0.292 mmol) in 2.5 mL of CH_2Cl_2 water (2.5 mL) H-cardanol azide **13** (0.157 g, 0.350 mmol), $CuSO_4.5H_2O$ (0.007g, 0.0292 mmol) and sodium ascorbate (0.010 g, 0.053 mmol) were added sequentially. Resulting mixture was allowed to stir at rt for three days. After completion of the reaction, the reaction mixture was diluted with CH_2Cl_2 (20 mL). Separated CH_2Cl_2 solution was washed with brine (10 mL), dried over Na_2SO_4, and solvent removed under reduced pressure to result in crude **8** as viscous solid. Upon purification by column chromatography using 30% EtOAc in hexanes as a eluent HTC **8** resulted in 94% yield (0.212 g) as colorless waxy solid. R_f = 0.5 (20% EtOAc in hexanes). IR Data (ν): 2956, 2919, 2850, 1620, 1604, 1582, 1497, 1455, 1355, 1250, 1049, 874, 750 cm^{-1}; ^1H NMR (400 MHz, $CDCl_3$) δ 7.83 (s, 1H), 7.31-7.26 (m, 2H), 7.14 (t, J = 7.6 Hz, 1H), 7.01-6.95 (q, 3H), 6.82-6.80 (d, 1H), 6.69-6.65 (dd, 2H), 5.22 (s, 2H), 4.76-4.74 (t, 2H), 4.35-4.33 (t, 2H), 2.58-2.54 (t, J, = 8 Hz, 2H), 1.61-1.57 (t, J = 7.6 Hz, 3H), 1.30-1.26 (m, 25H), 0.90-0.87 (t, 3H) ppm; ^{13}C NMR (100 MHz, $CDCl_3$) δ 158.3, 157.8, 145.0, 129.6, 129.5, 124.0, 122.0, 121.3, 114.9, 114.8, 111.5, 66.2, 62.0, 50.0, 36.1, 32.0, 31.5, 29.87, 29.83, 29.7, 29.6, 22.8, 14.2 ppm. HRMS (ESI) Calcd for $C_{32}H_{47}N_3O_2$ [M+H] 506.3747amu, found 506.3742 amu.

2. Table of contents

IR (KBr) ν_{max}, ^{1}H NMR (400 MHz, $CDCl_3$), ^{13}C NMR (100 MHz, $CDCl_3$) and DEPT–135 spectra of Phenyl propargyl ether. S5, S6

IR (KBr) ν_{max}, ^{1}H NMR (400 MHz, $CDCl_3$), ^{13}C NMR (100 MHz, $CDCl_3$), DEPT–135 and HRMS spectra of 4-(Propargyloxy)-benzaldehyde **9**. S7- S9

IR (KBr) ν_{max}, ^{1}H NMR (400 MHz, $CDCl_3$), ^{13}C NMR (100 MHz, $CDCl_3$), DEPT–135 and HRMS spectra of 1-(2-Bromoethoxy)-3-pentadecylbenzene. S9- S11

IR (KBr) ν_{max}, ^{1}H NMR (400 MHz, $CDCl_3$), ^{13}C NMR (100 MHz, $CDCl_3$), DEPT–135 and HRMS spectra of H-Cadanolazide **13**. S12- S14

IR (KBr) ν_{max}, ^{1}H NMR (400 MHz, $CDCl_3$), ^{13}C NMR (100 MHz, DMSO - d_6), DEPT–135 and HRMS spectra of 5,10,15,20-Tetra(4-propargyloxyphenyl)porphyrin **12**. S14- S16

IR (KBr) ν_{max}, ^{1}H NMR (400 MHz, $CDCl_3$), ^{13}C NMR (100 MHz, $CDCl_3$), DEPT–135 and HRMS spectra 5,10,15,20-Tetra(4-methoxyphenyl) porphyrin Zn **7**. S17- S19

IR (KBr) ν_{max}, ^{1}H NMR (400 MHz, $CDCl_3$), ^{13}C NMR (100 MHz, $CDCl_3$), DEPT–135 and HRMS spectra of H-cardanol triazole porphyrin conjugate (HTZPC) **5**. S19- S21

IR (KBr) ν_{max}, ^{1}H NMR (400 MHz, DMSO - d_6), ^{13}C NMR (100 MHz, $CDCl_3$) and HRMS spectra of Triazole Zinc Porphyrin Conjugate (TZPC) **6** S22, S23

IR (KBr) ν_{max}, ^{1}H NMR (400 MHz, $CDCl_3$), ^{13}C NMR (100 MHz, $CDCl_3$), DEPT–135 and HRMS spectra of 1-(2-(3-pentadecylphenoxy)ethyl-4-(phenoxymethyl)-1*H*-1,2,3-triazole (HTC) **8**. S24- S26

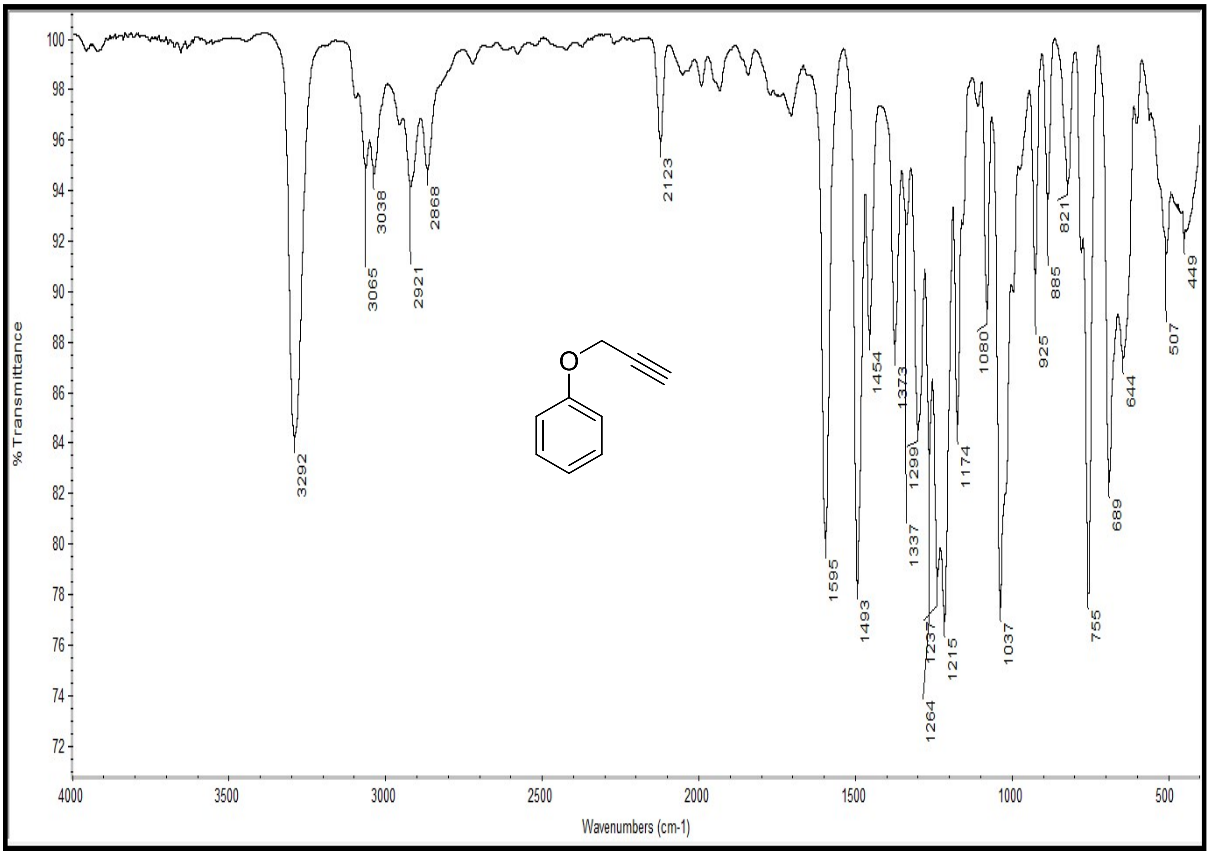

IR spectrum of Phenyl propargyl ether**.**

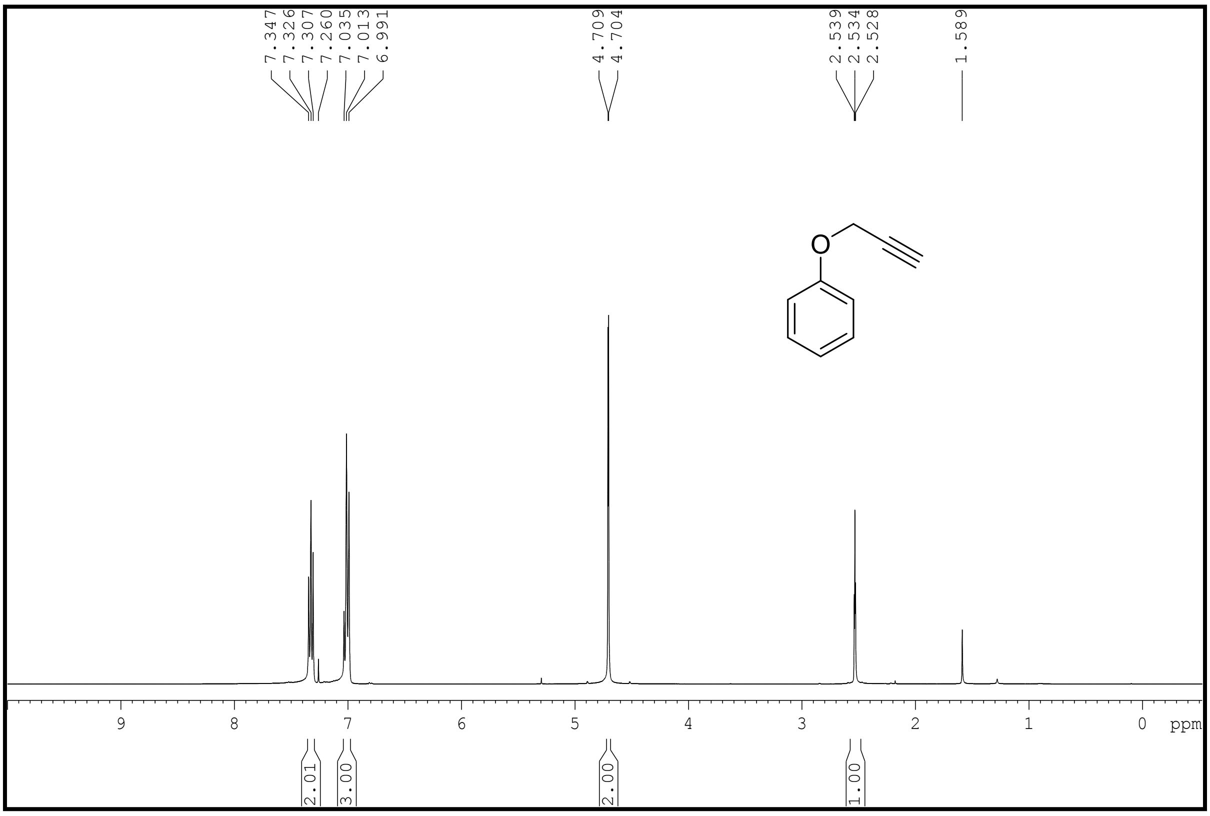

^{1}H NMR (400 MHz, $CDCl_3$) spectrum of Phenyl propargyl ether

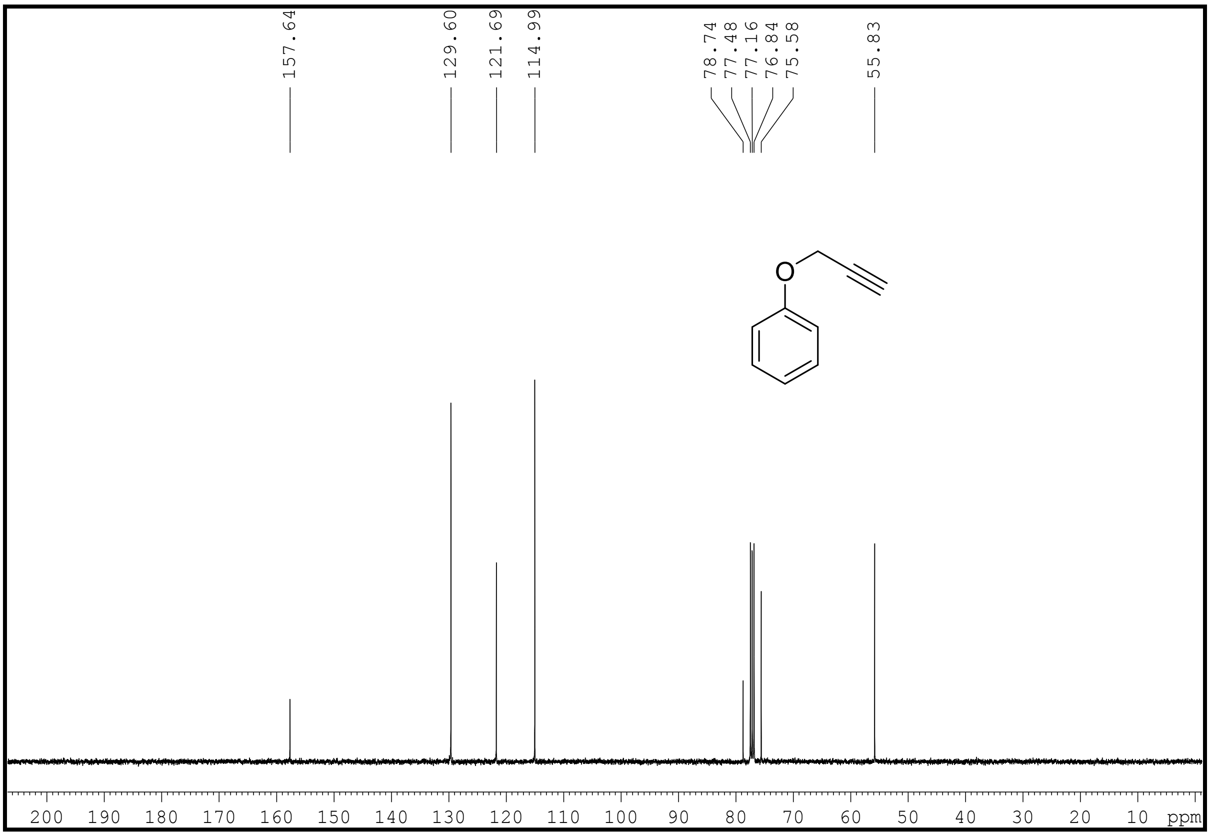

^{13}C NMR (100 MHz, $CDCl_3$) spectrum of Phenyl propargyl ether.

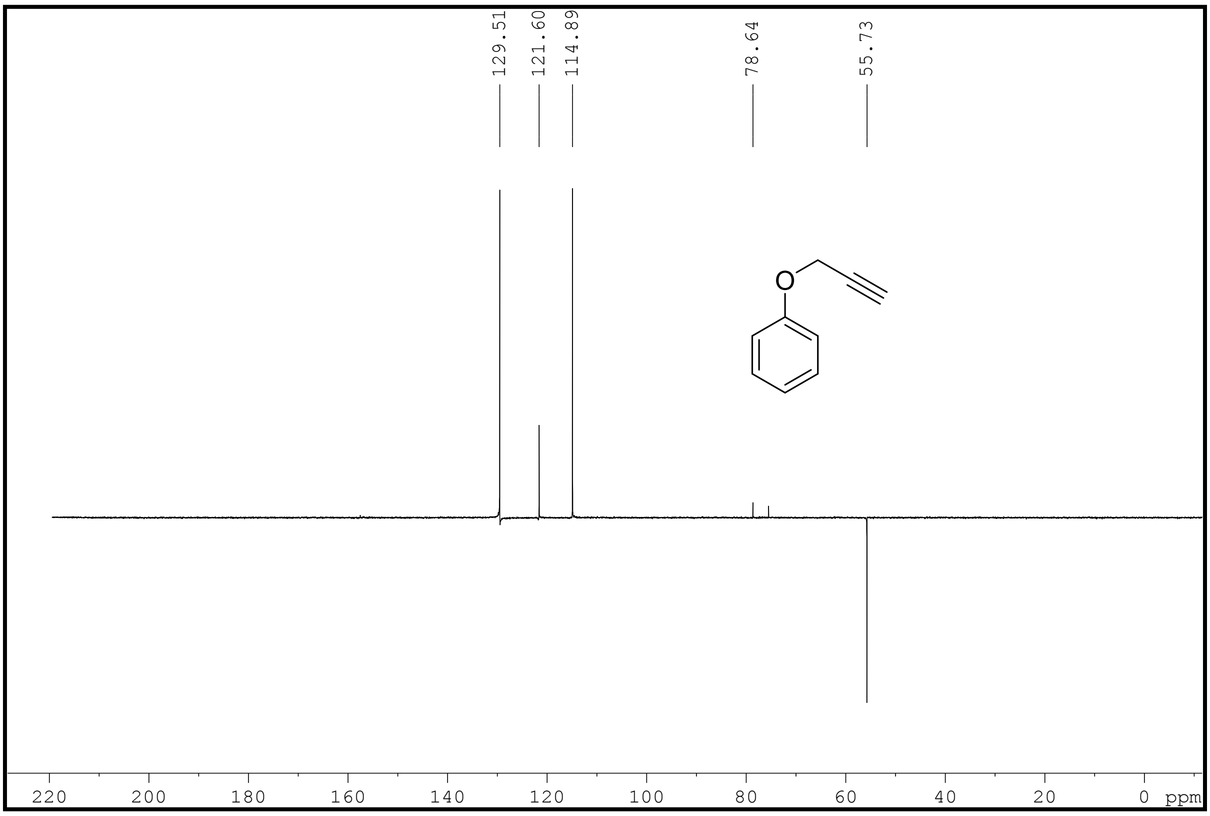

DEPT -135 NMR spectra of Phenyl propargyl ether.

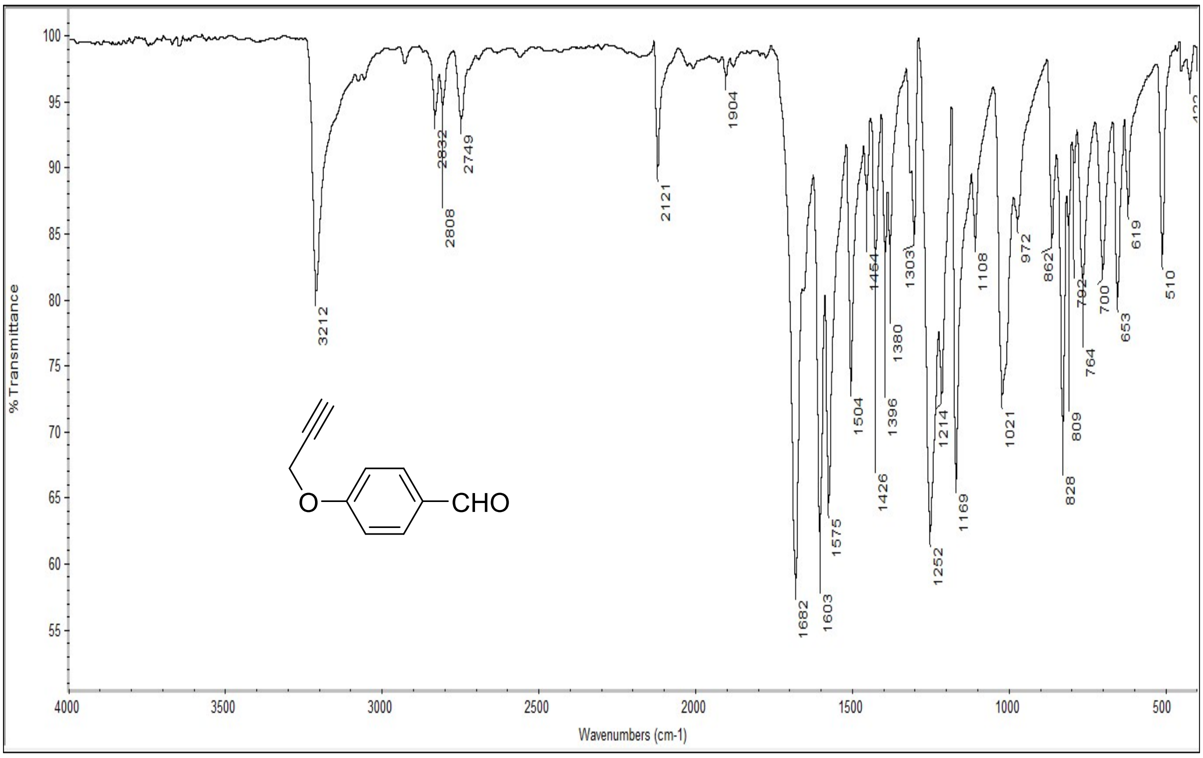

IR (KBr) ν_{max} spectrum of 4-(Propargyloxy)benzaldehyde **9.**

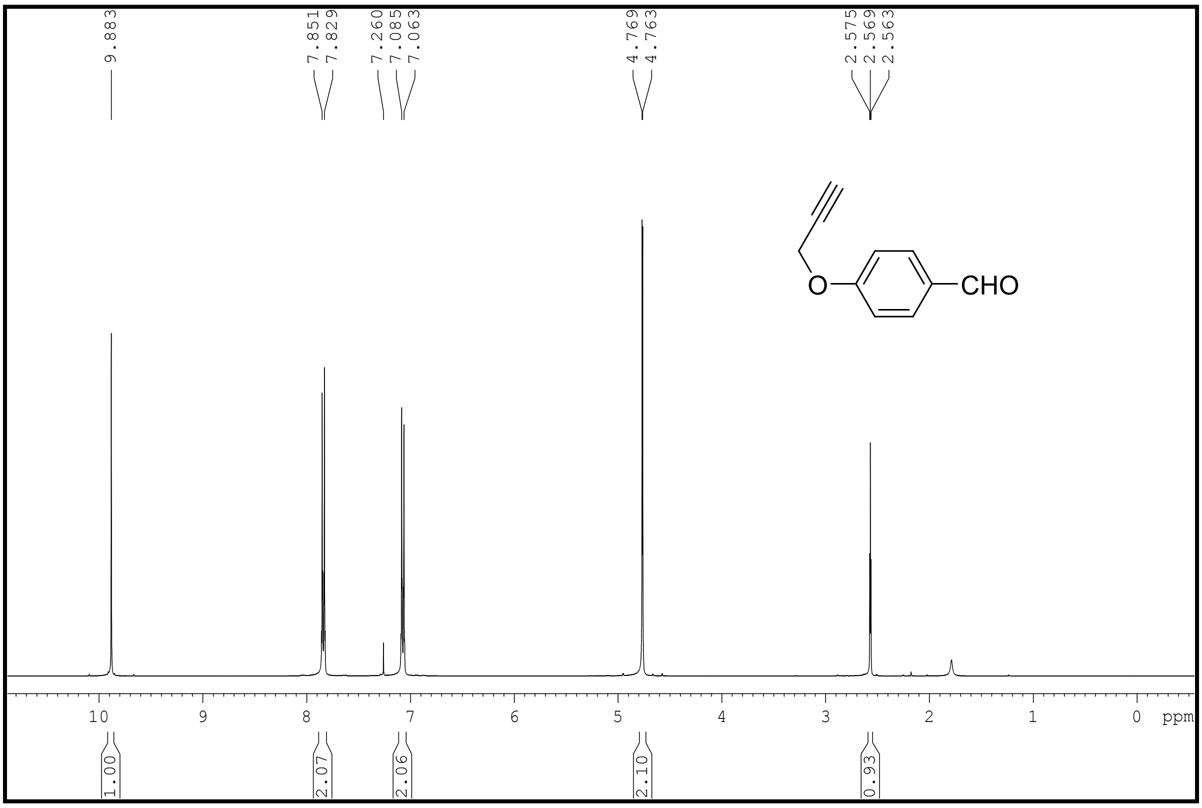

^{1}H NMR (400 MHz, $CDCl_3$) spectrum of 4-(Propargyloxy)benzaldehyde **9.**

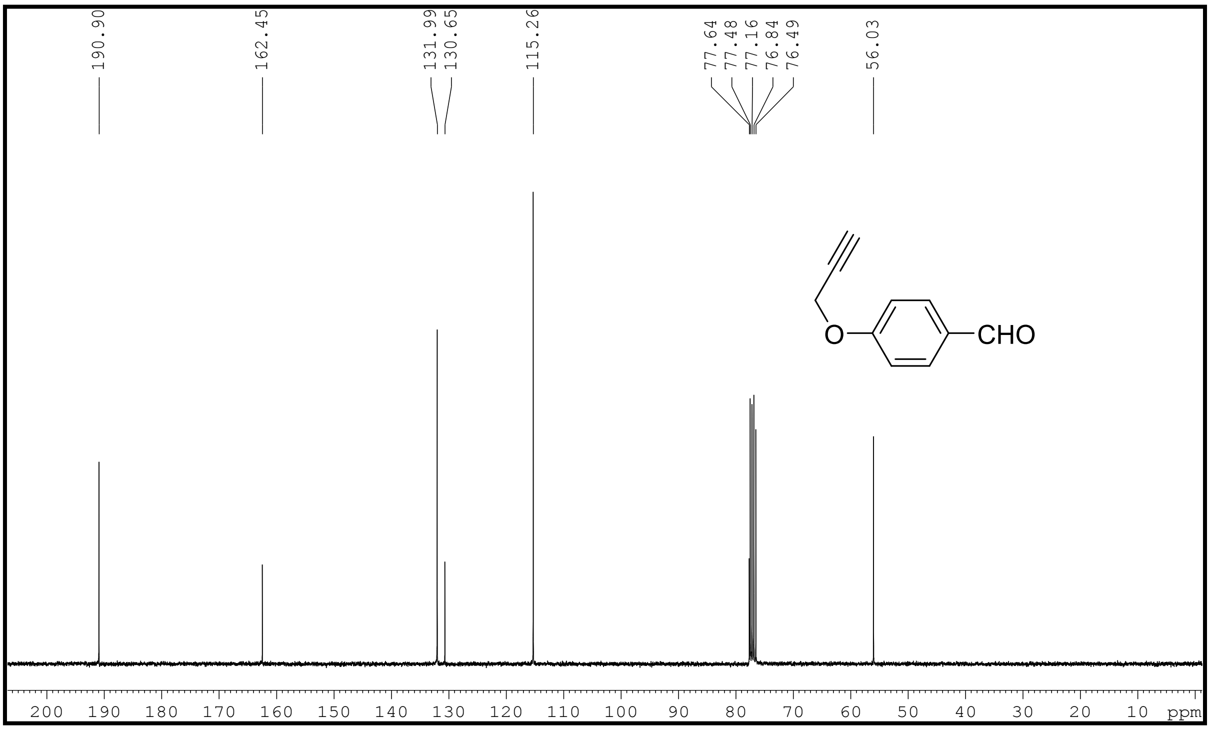

^{13}C NMR (100 MHz, $CDCl_3$) spectrum of 4-(Propargyloxy)benzaldehyde **9.**

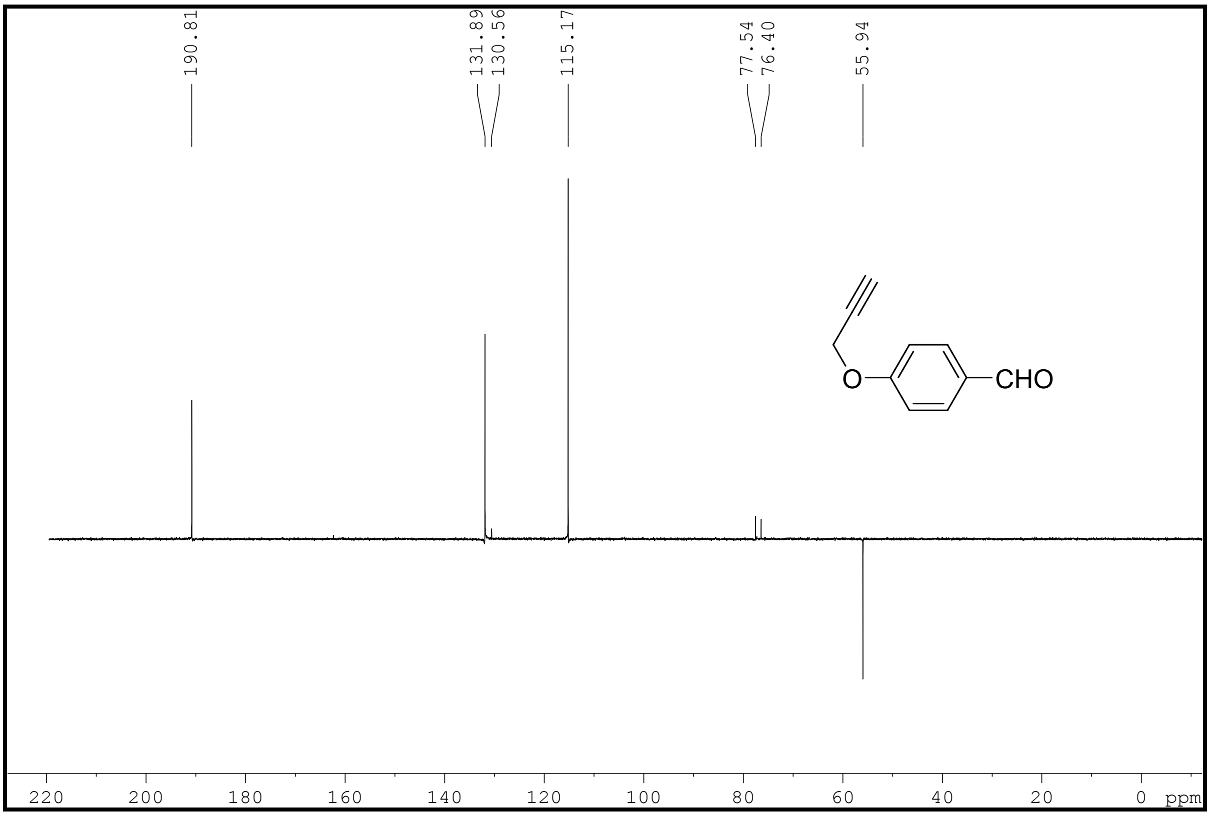

DEPT-135 NMR spectrum of 4-(Propargyloxy)-benzaldehyde **9**.

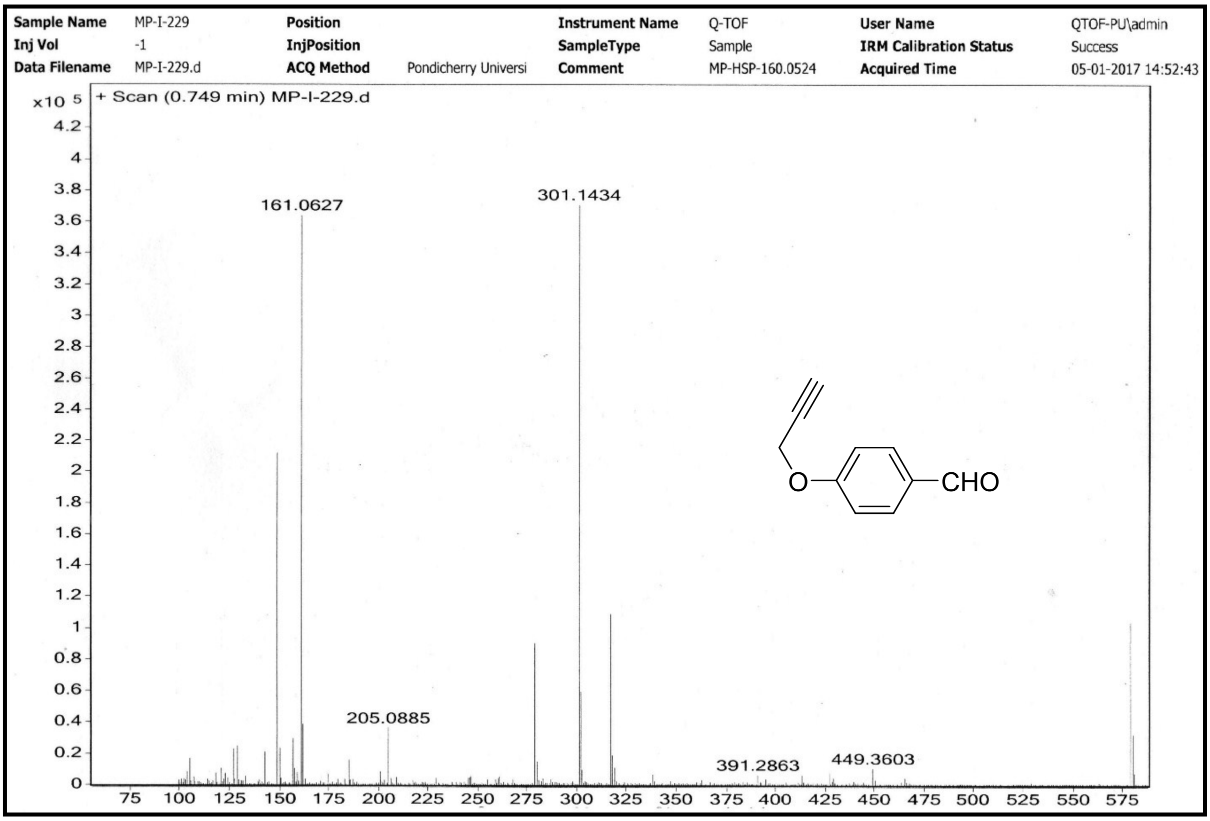

HRMS spectrum of 4-(Propargyloxy)benzaldehyde **9.**

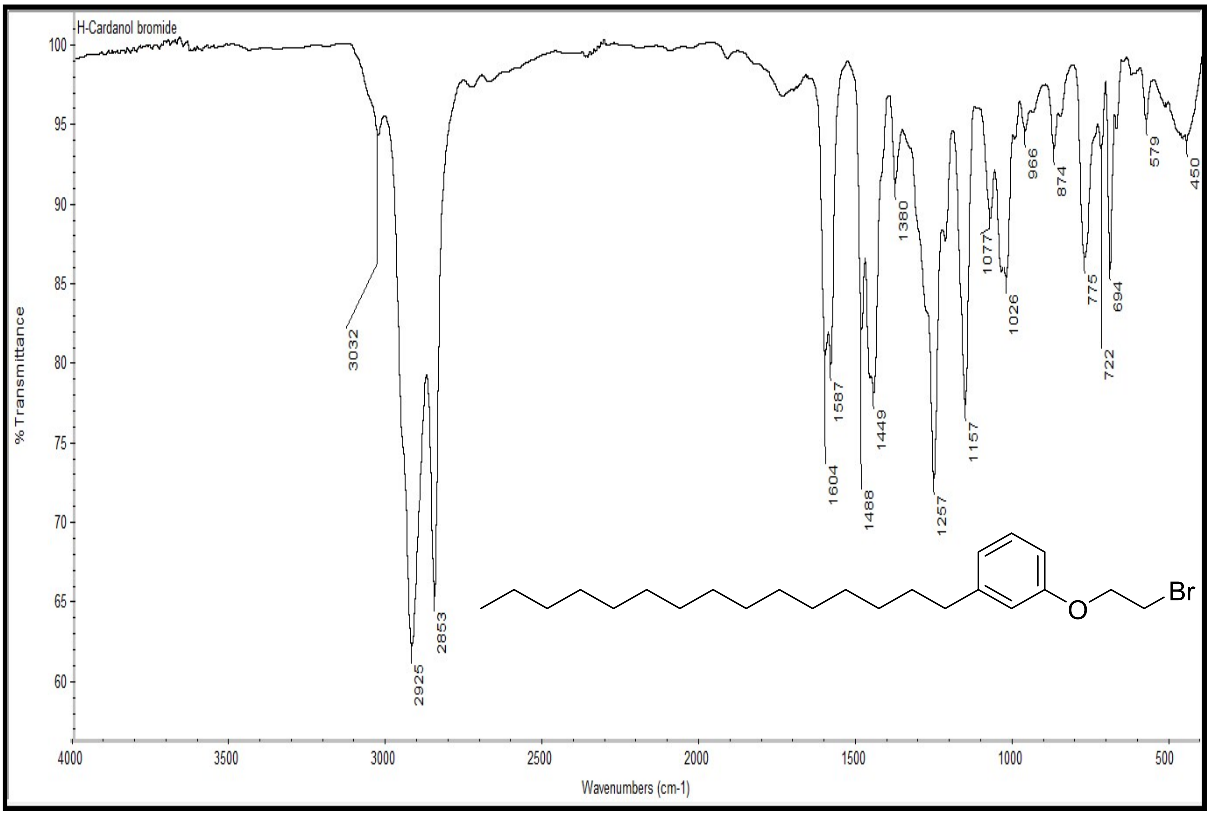

IR (KBr) ν_{max} spectrum of 1-(2-Bromoethoxy)-3-pentadecylbenzene.

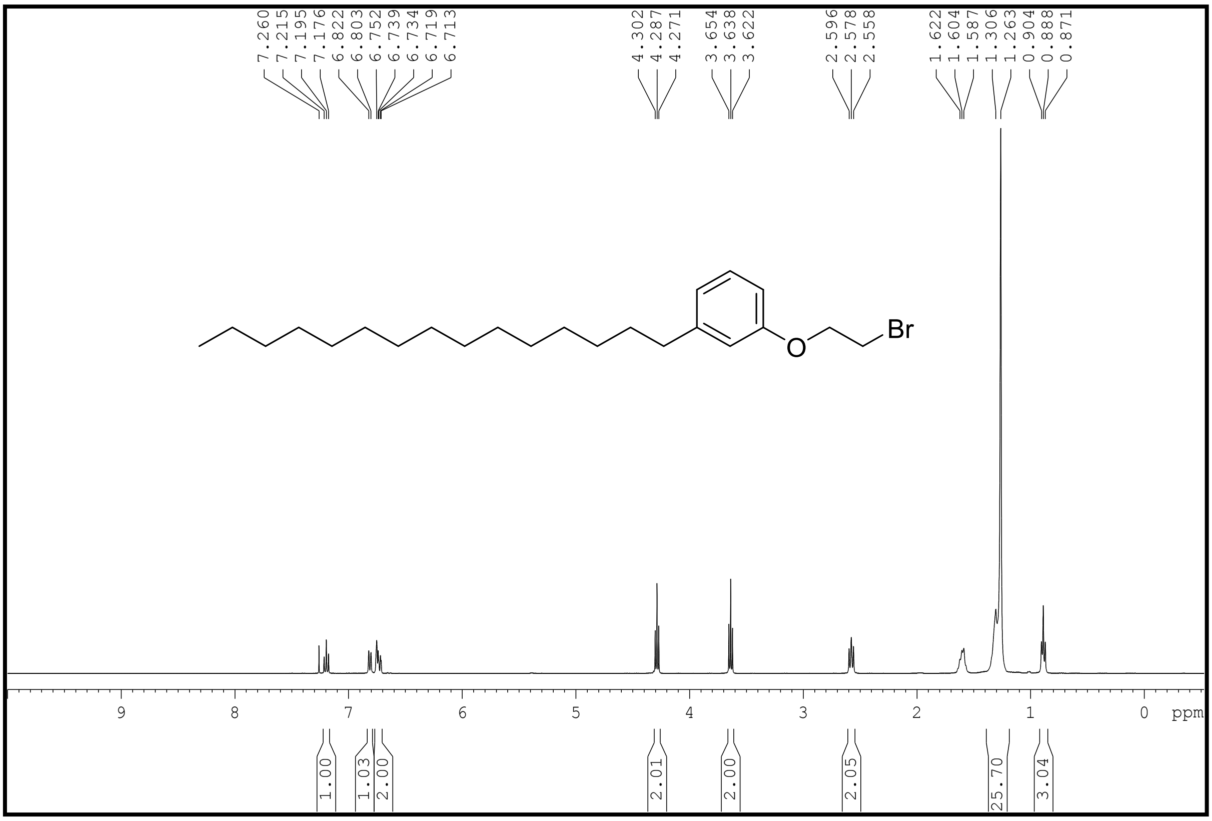

^{1}H NMR (400 MHz, $CDCl_3$) spectrum of 1-(2-Bromoethoxy)-3-pentadecylbenzene.

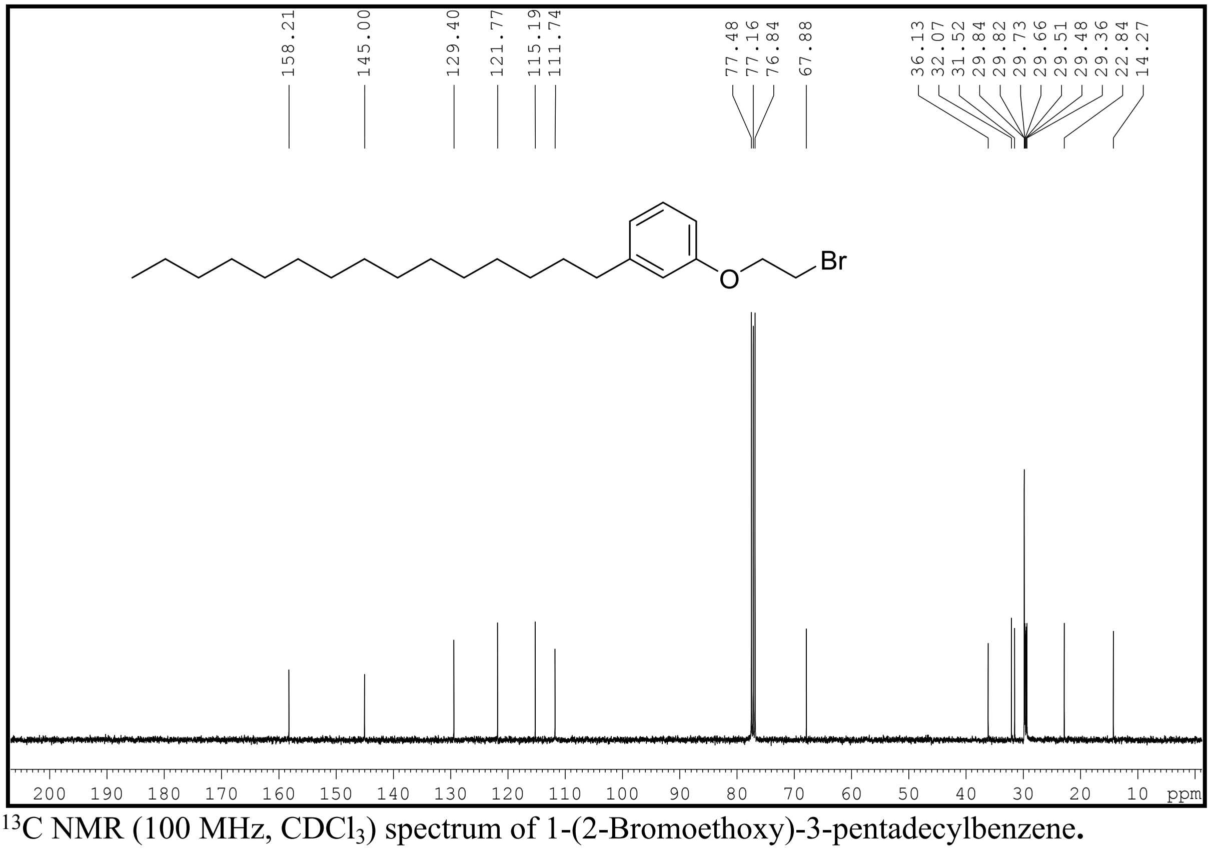

^{13}C NMR (100 MHz, $CDCl_3$) spectrum of 1-(2-Bromoethoxy)-3-pentadecylbenzene.

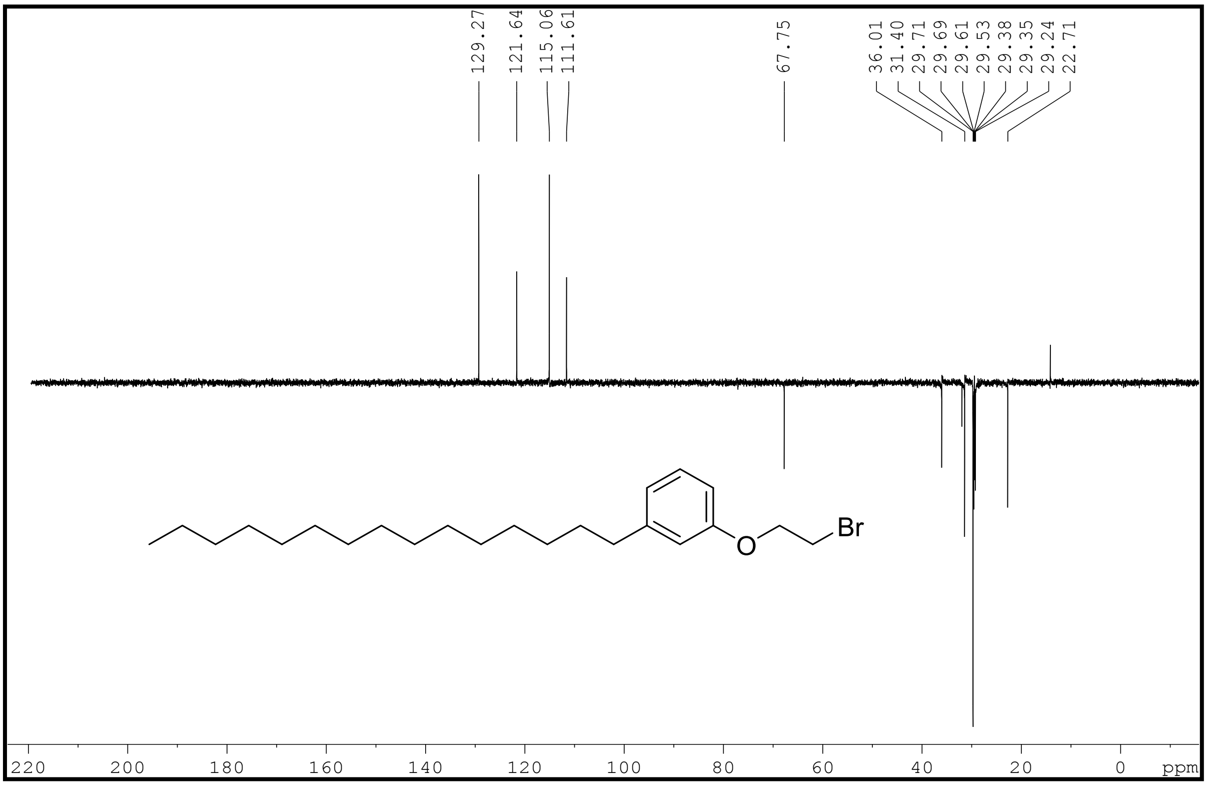

DEPT-135 NMR spectrum of 1-(2-Bromoethoxy)-3-pentadecylbenzene.

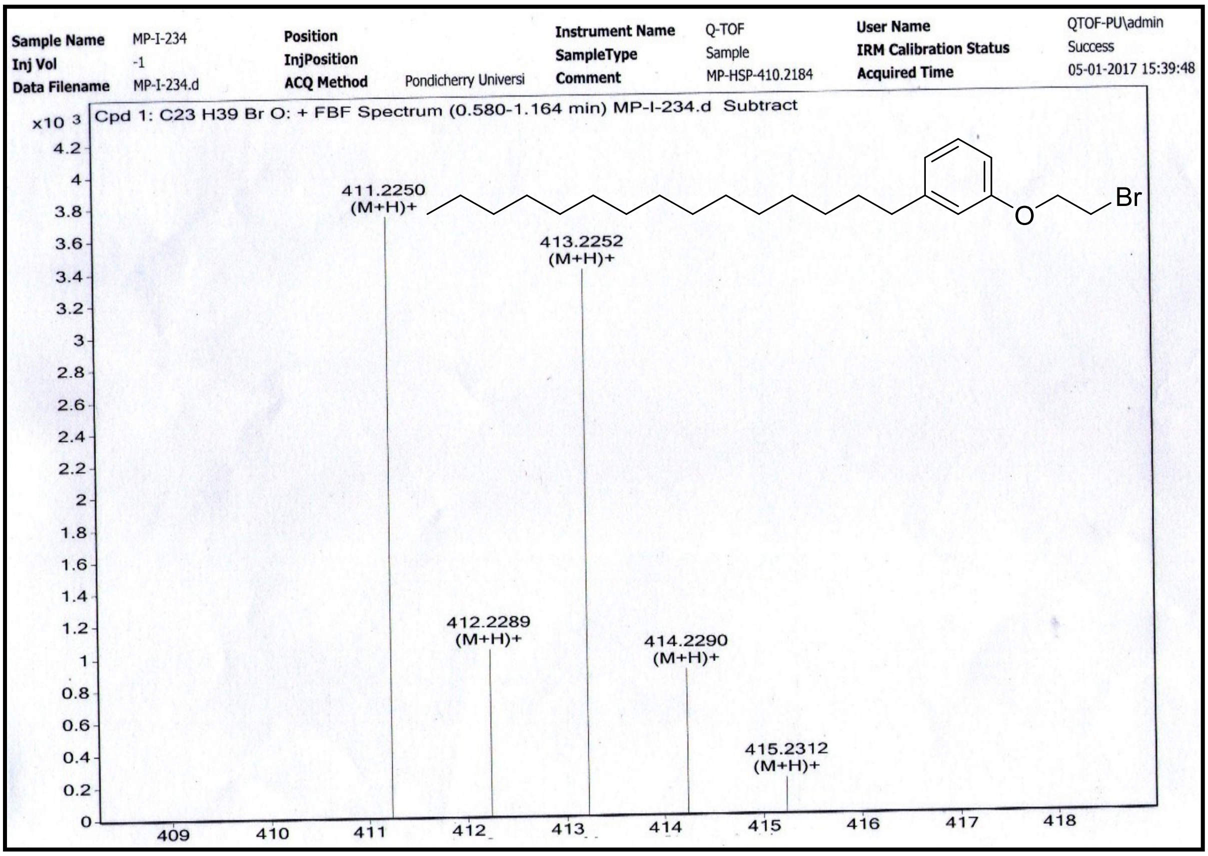

HRMS spectrum of 1-(2-Bromoethoxy)-3-pentadecylbenzene.

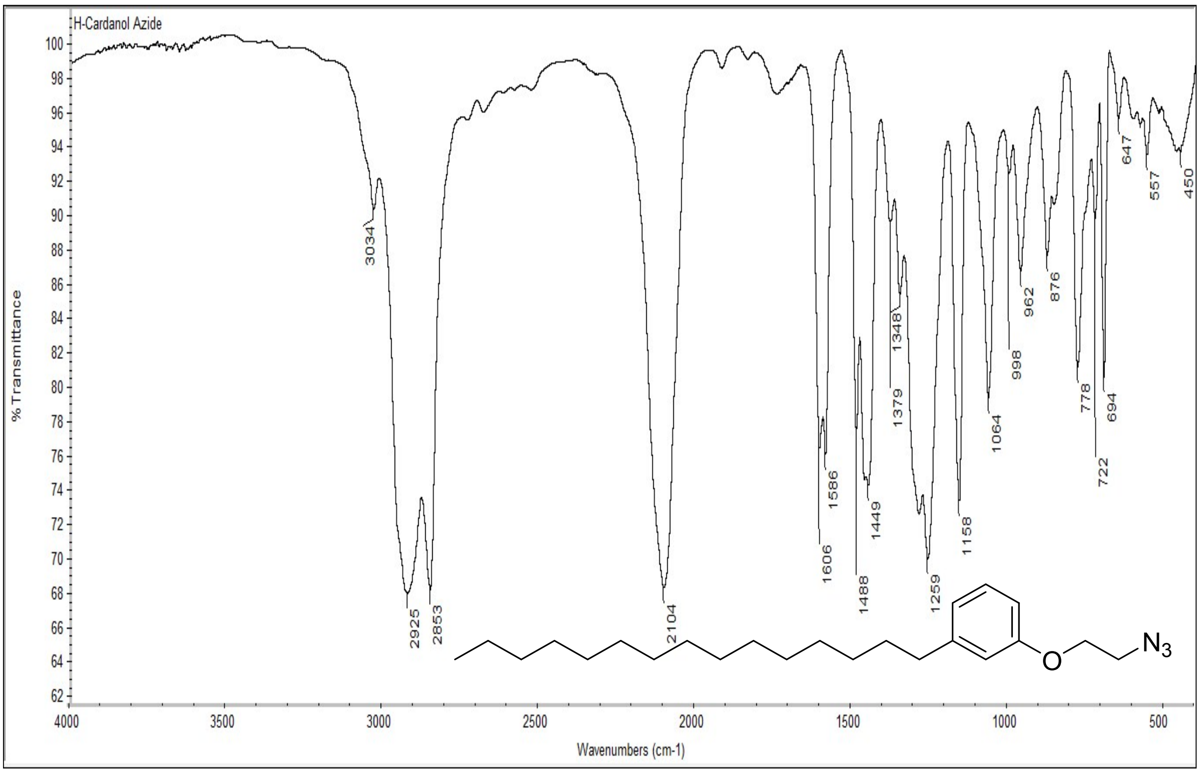

IR (KBr) ν_{max} spectrum of H-Cardanol azide **13.**

.

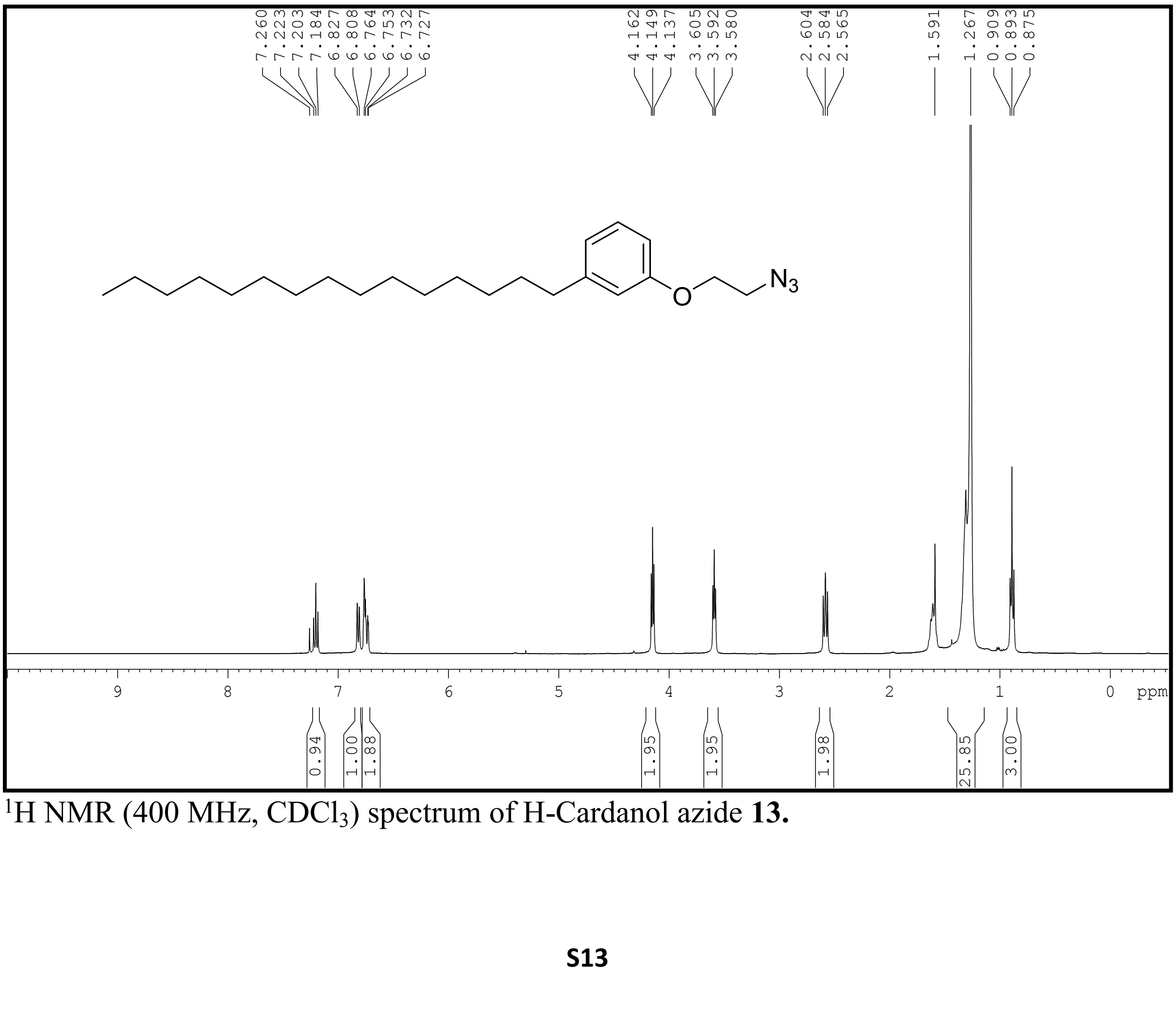

^{1}H NMR (400 MHz, $CDCl_3$) spectrum of H-Cardanol azide **13.**

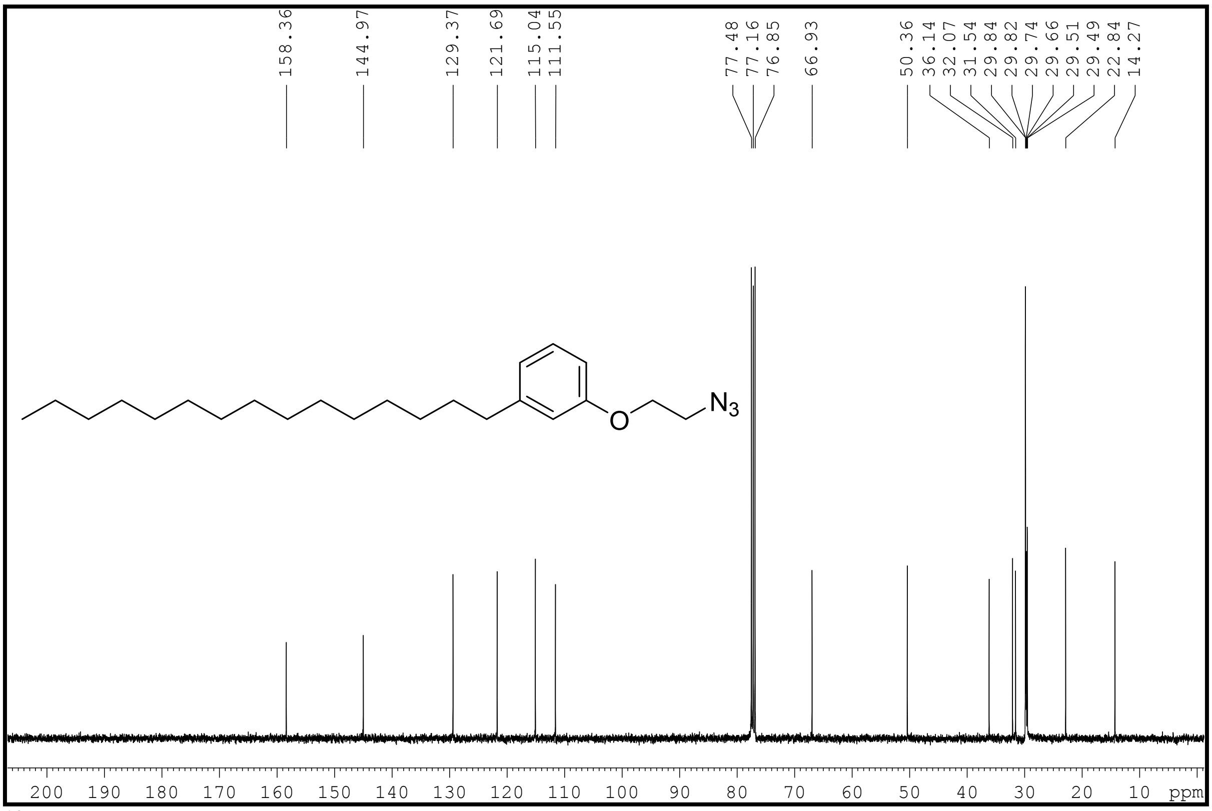

^{13}C NMR (100 MHz, $CDCl_3$) spectrum of H-Cardanol azide **13.**

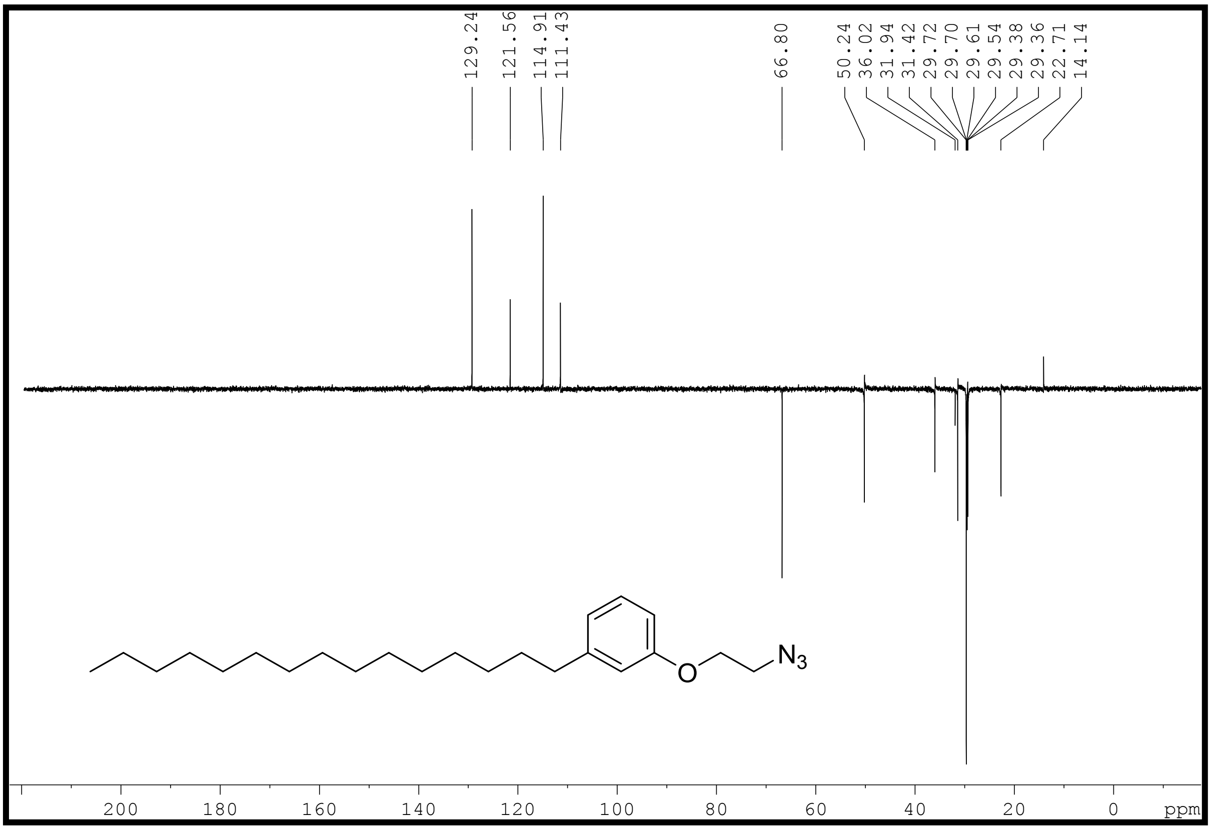

DEPT-135 NMR spectrum of H-cardanol azide **13.**

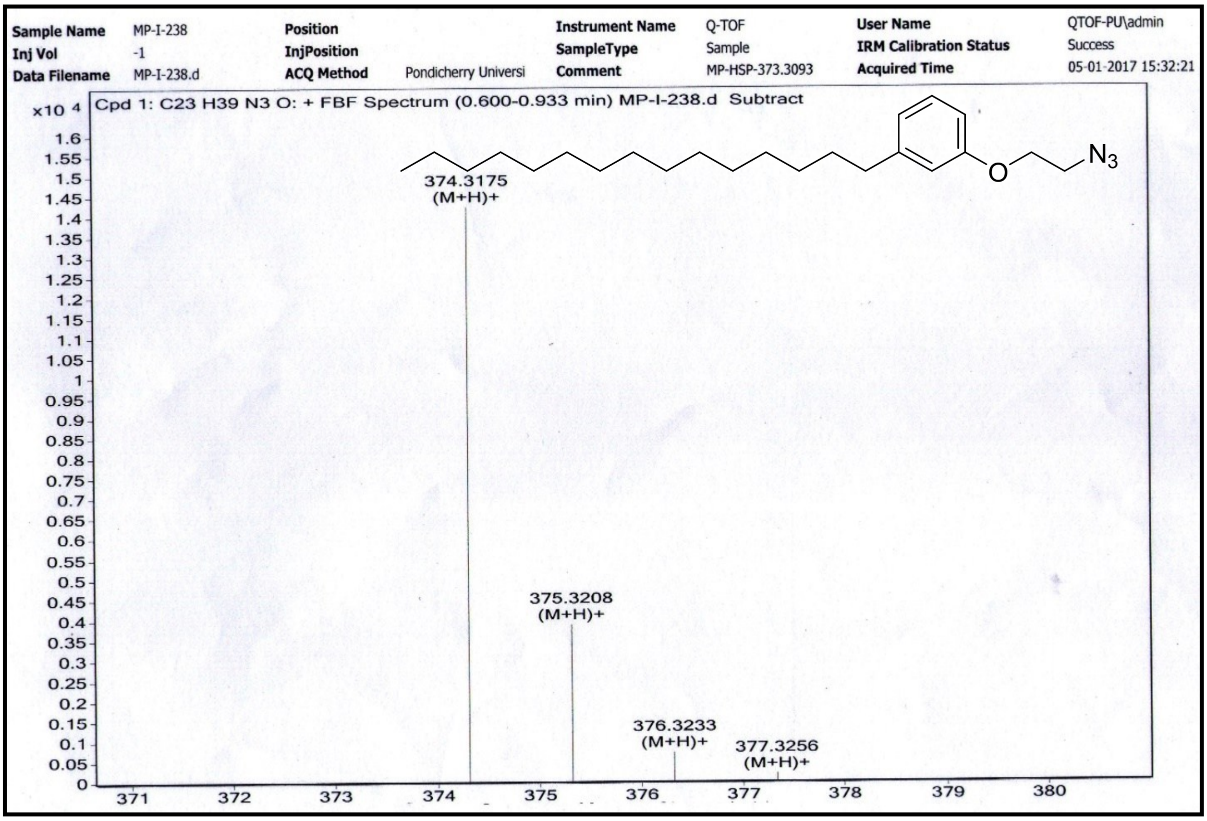

HRMS spectrum of H-Cardanol azide **13.**

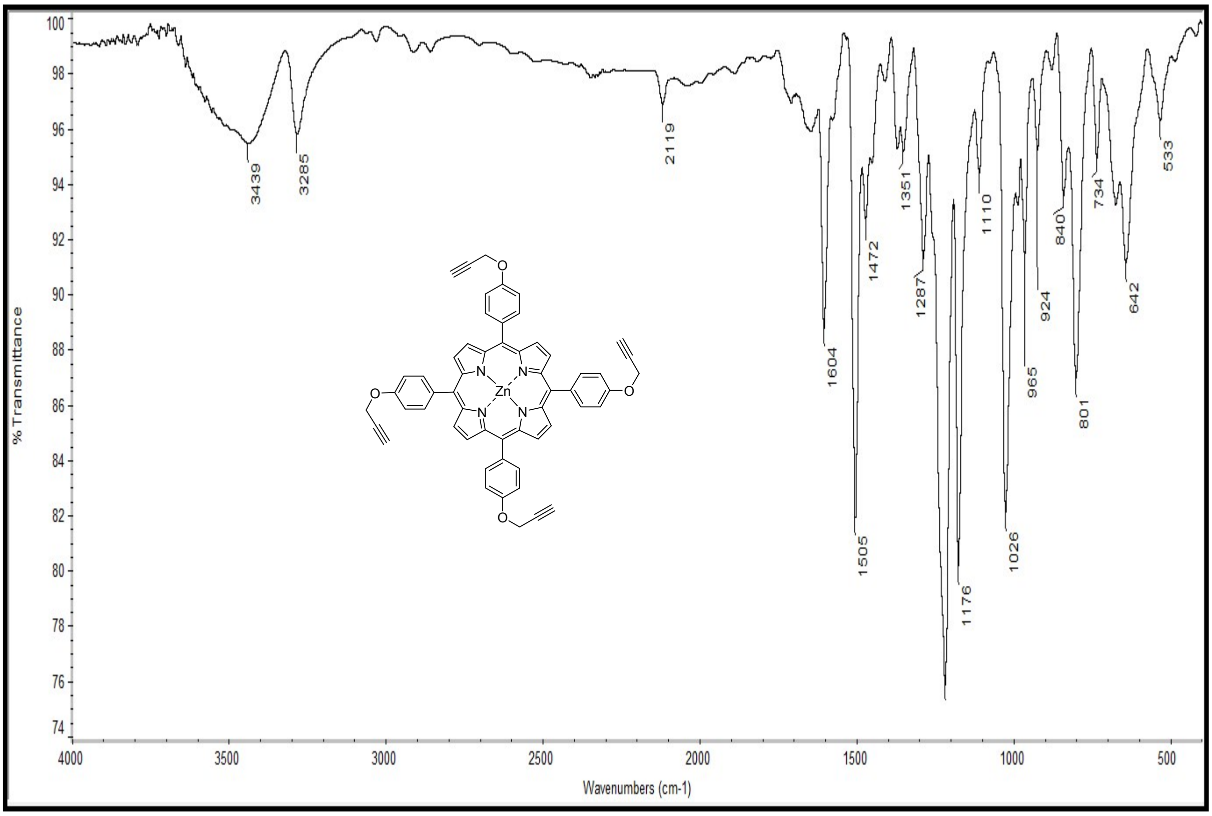

IR (KBr) ν_{max} spectrum of 5,10,15,20-Tetra(4-propargyloxyphenyl)porphyrin Zn **12.**

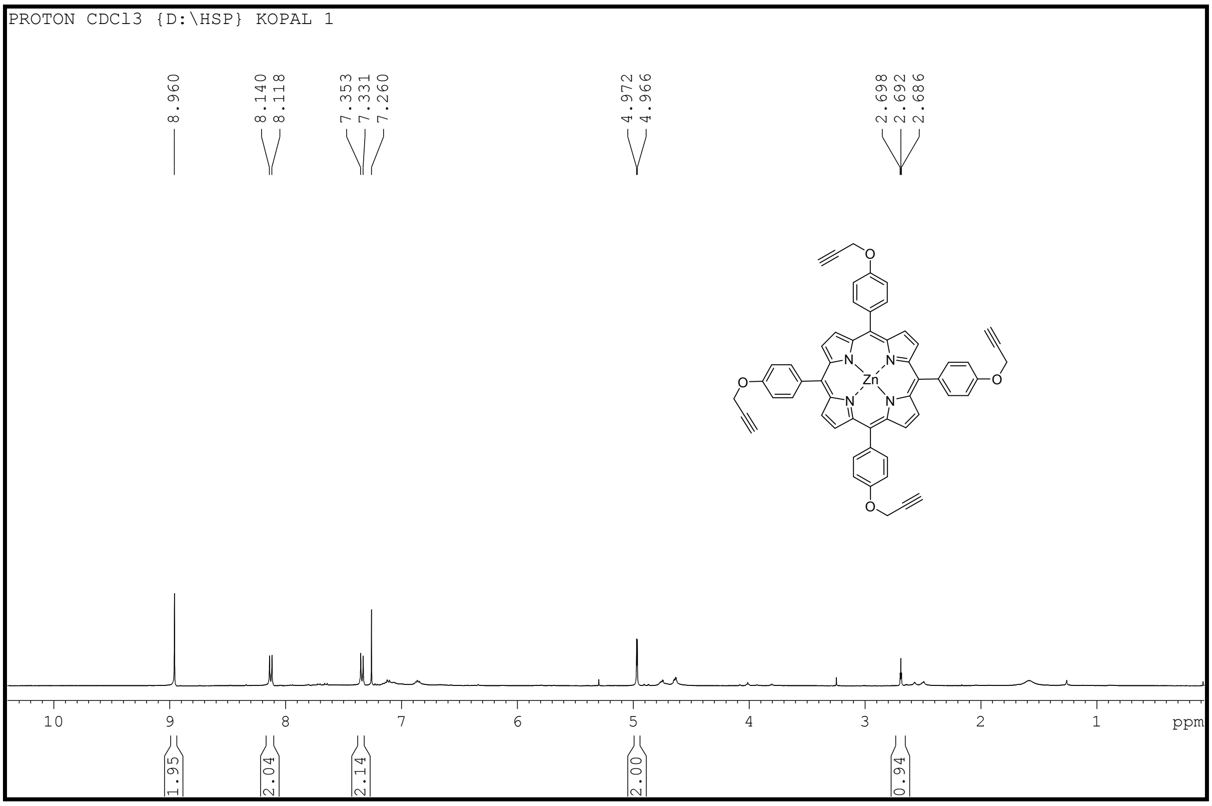

^{1}H NMR (400 MHz, $CDCl_3$) spectrum of 5,10,15,20-Tetra(4-propargyloxyphenyl)porphyrin Zn **12**.

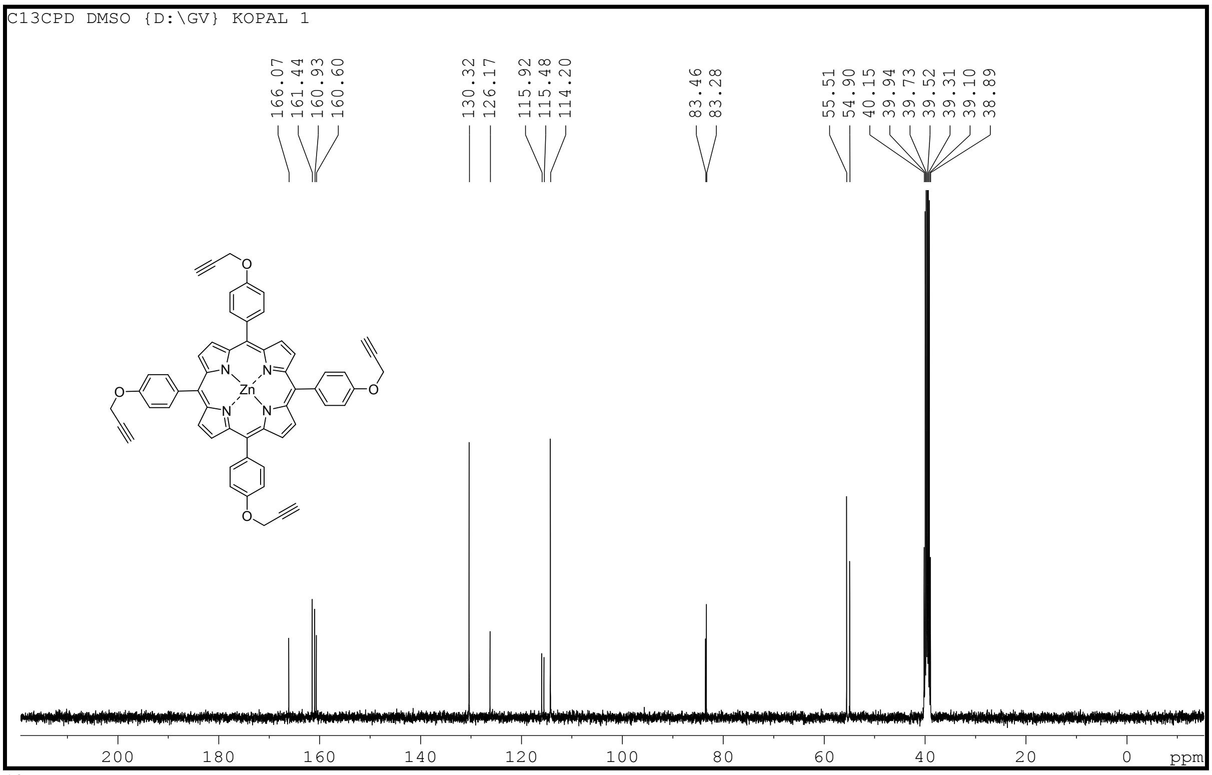

^{13}C NMR (100 MHz, DMSO - d_6) spectrum 5,10,15,20-tetra(4-propargyloxyphenyl)porphyrin Zn **12.**

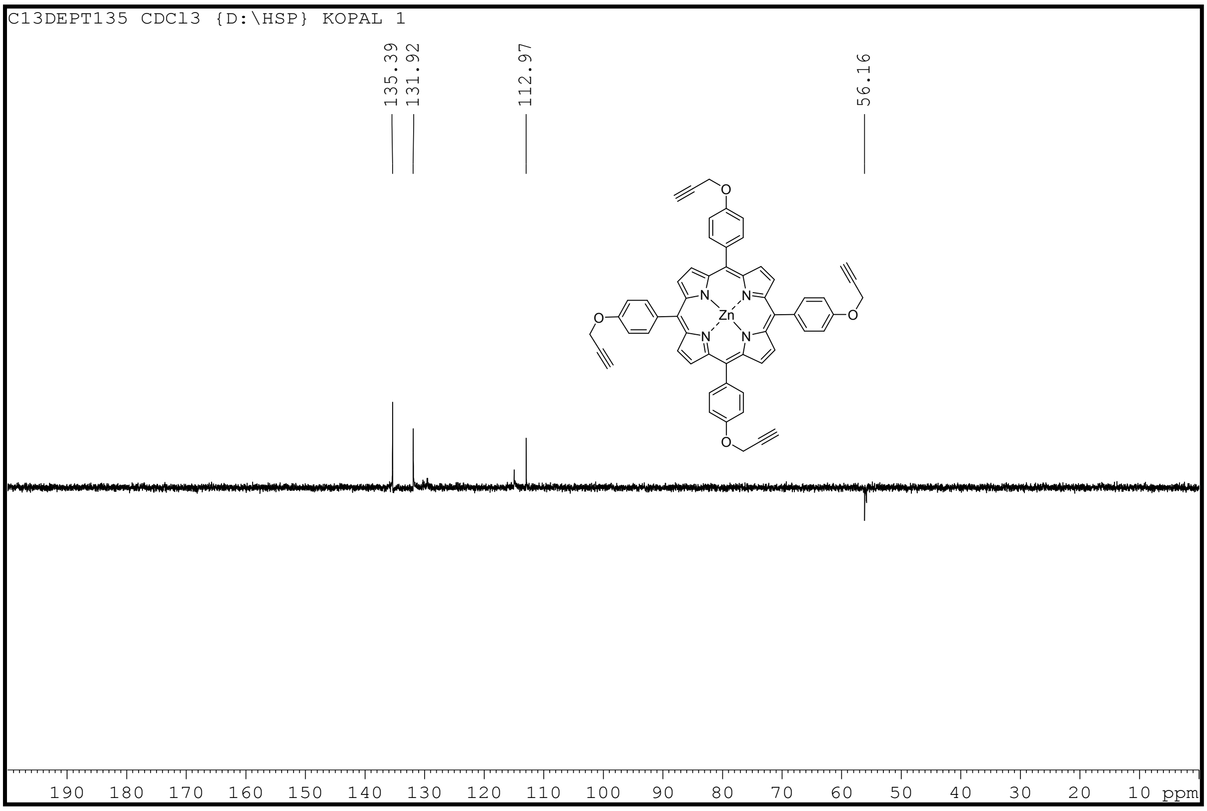

DEPT-135 NMR spectrum of 5,10,15,20-Tetra(4-propargyloxyphenyl)porphyrin Zn **12.**

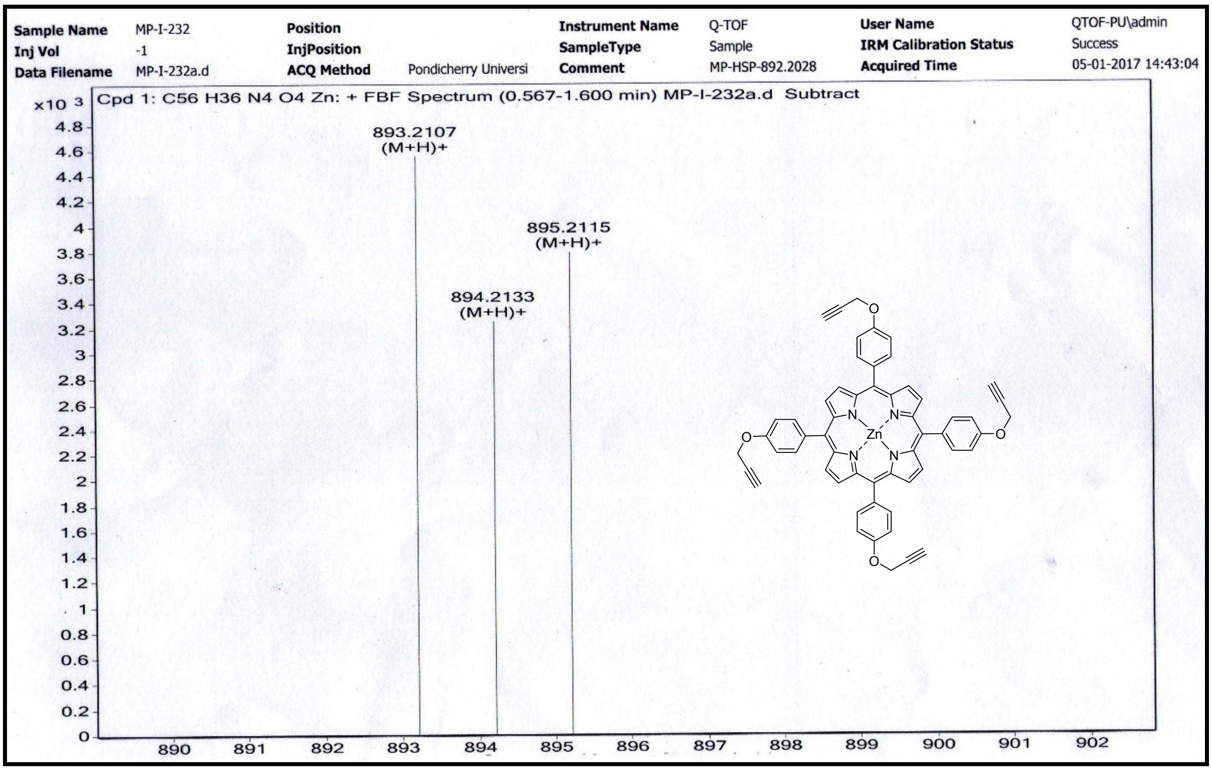

HRMS spectrum of 5,10,15,20-Tetra(4-propargyloxyphenyl)porphyrin Zn **12.**

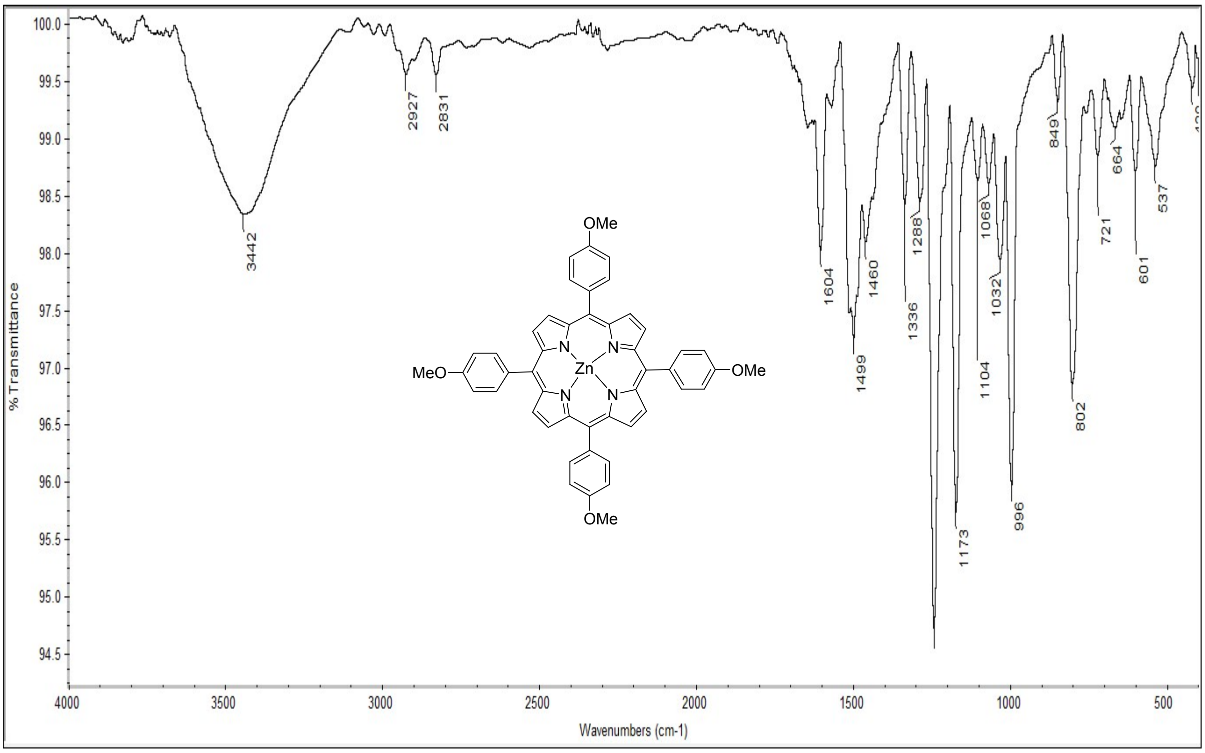

IR (KBr) ν_{max} spectrum of 5,10,15,20-Tetra(4-methoxyphenyl) porphyrin Zn **7**.

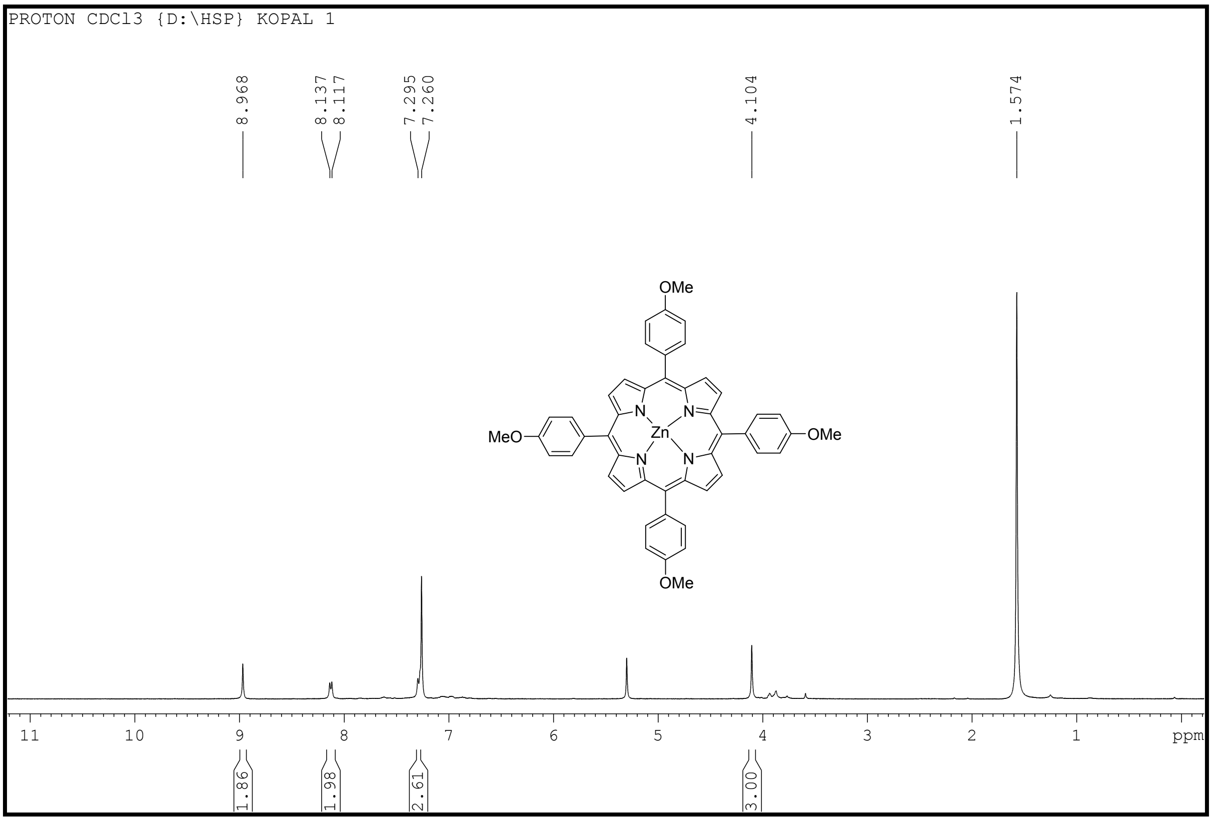

^{1}H NMR (400 MHz, $CDCl_3$) spectrum of 5,10,15,20-Tetra(4-methoxyphenyl) porphyrin Zn **7.**

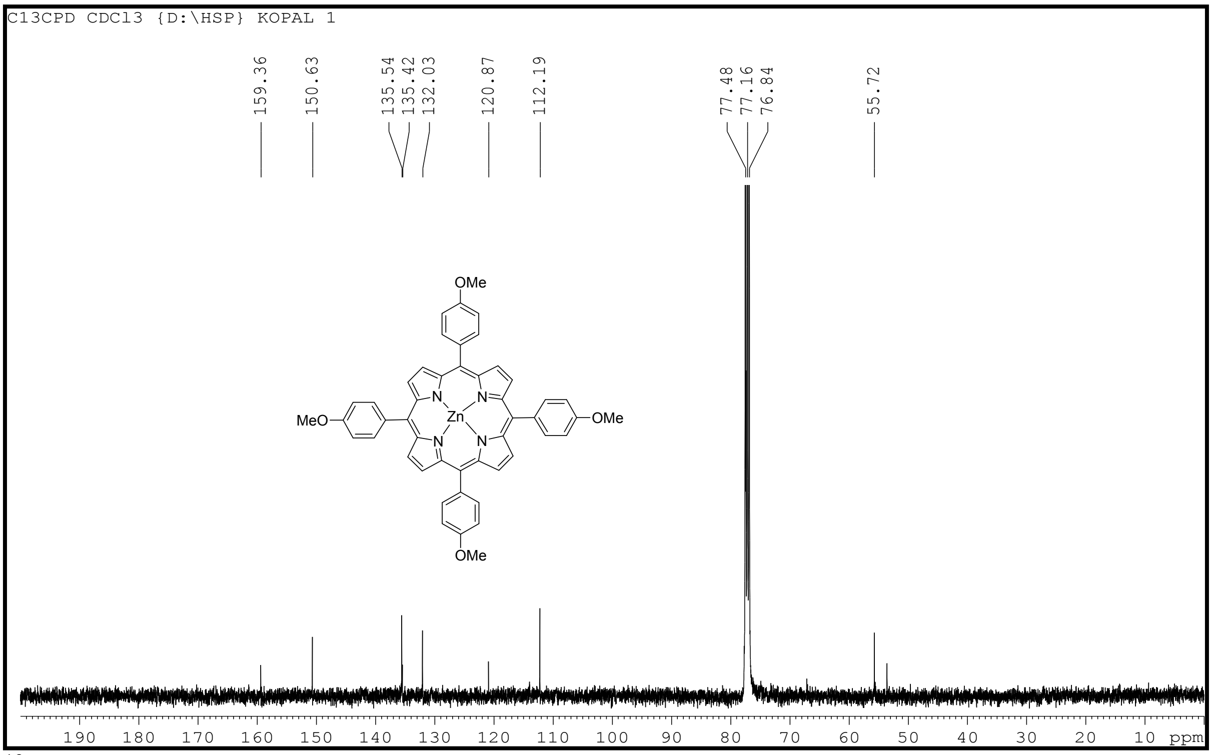

^{13}C NMR (100 MHz, $CDCl_3$) spectrum of 5,10,15,20-tetra(4-methoxyphenyl) porphyrin Zn **7**.

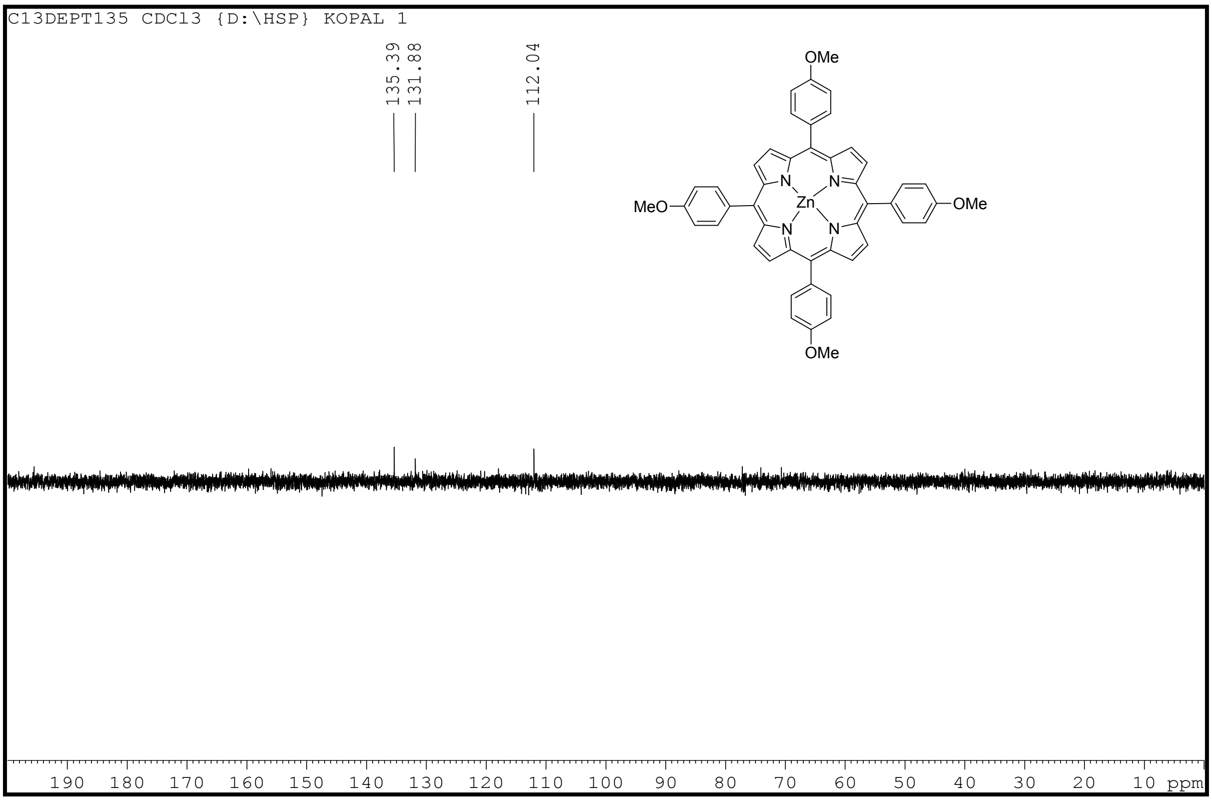

DEPT- 135 NMR spectrum of 5,10,15,20-tetra(4-methoxyphenyl)porphyrin Zn **7.**

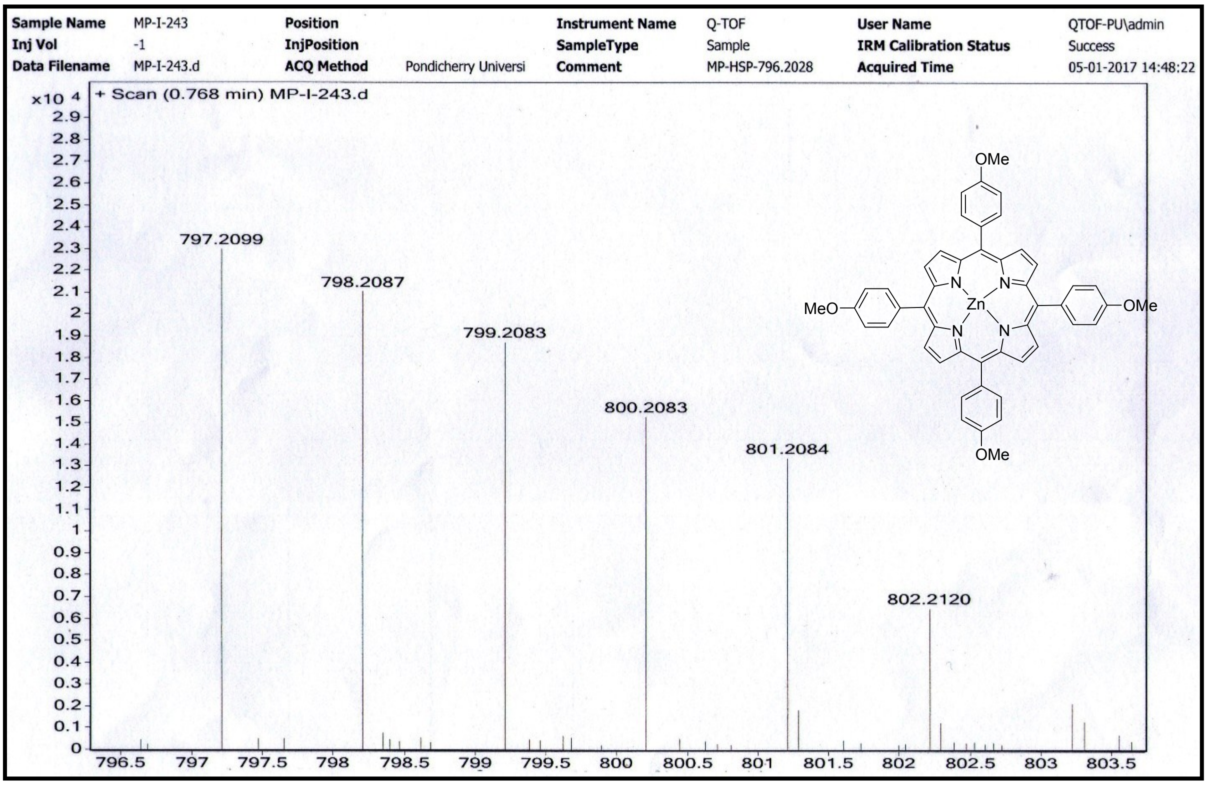

HRMS spectrum of 5,10,15,20-Tetra(4-methoxyphenyl) porphyrin Zn **7**.

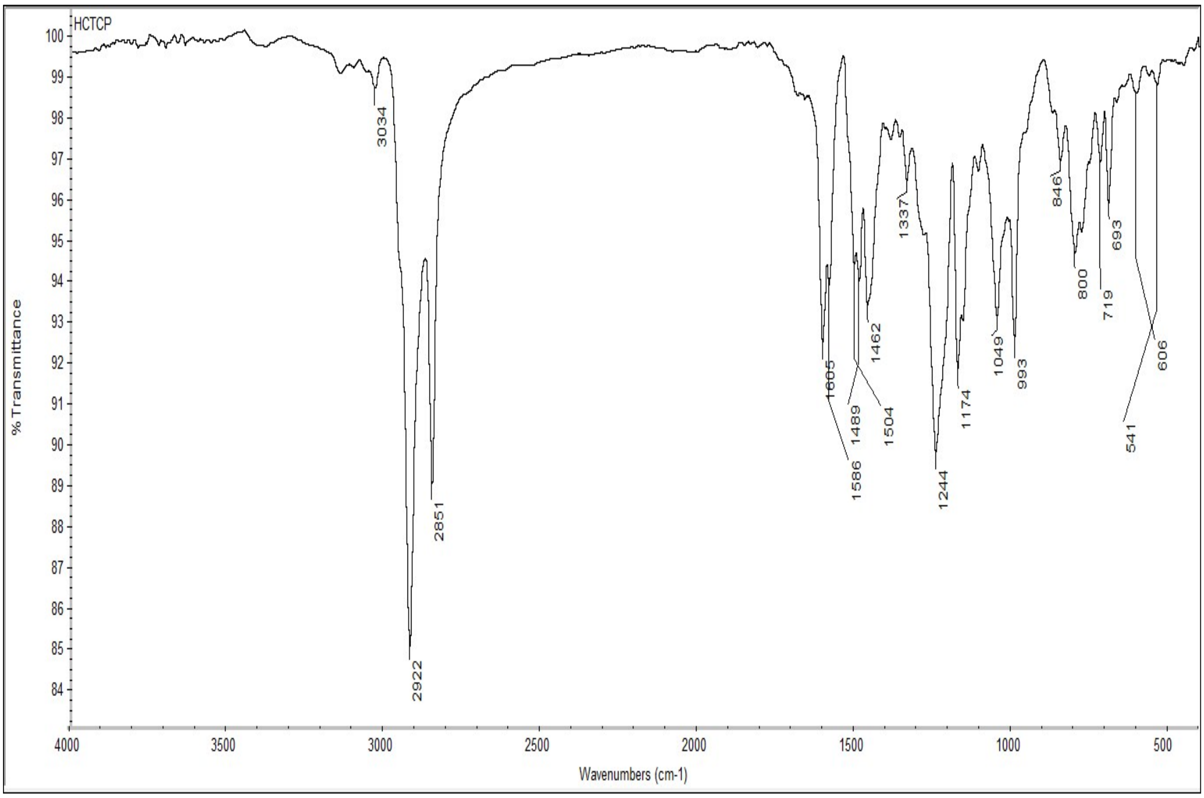

IR (KBr) ν_{max} spectrum of hydrogenated cardanol triazole zinc porphyrin conjugate (HTZPC) **5**.

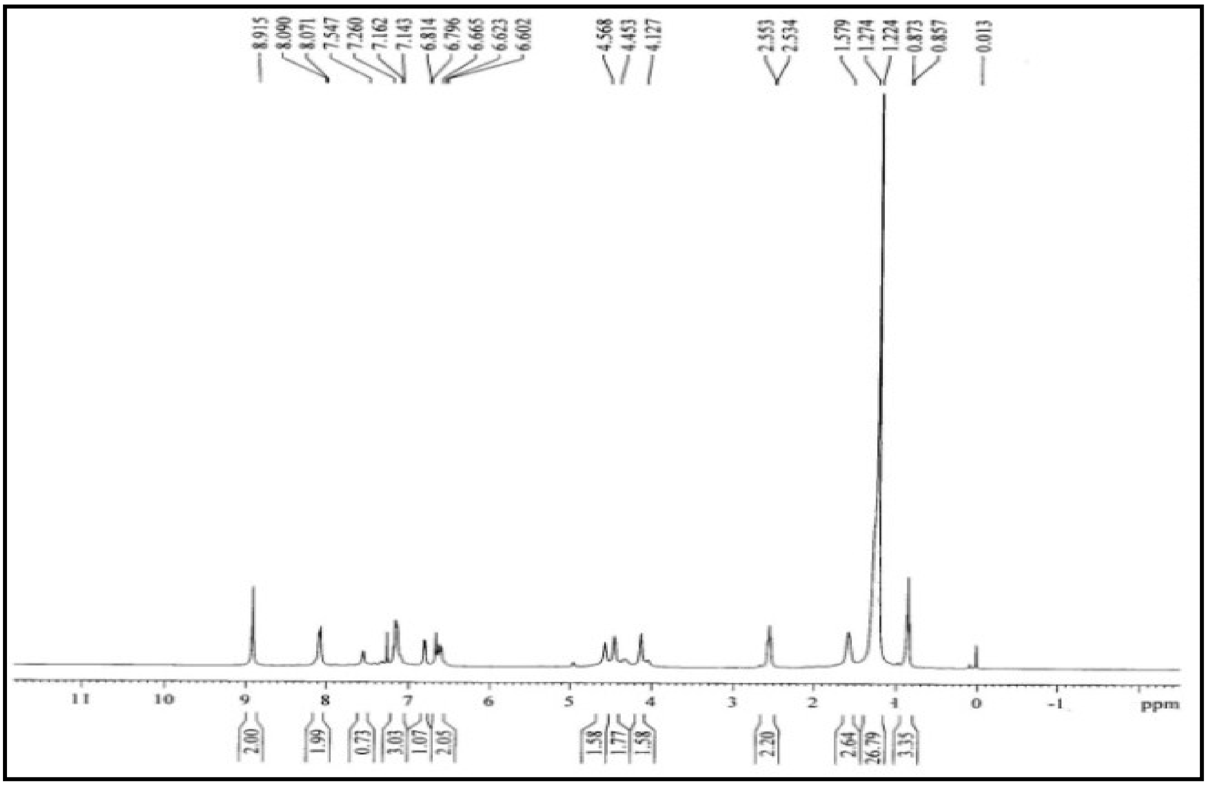

^{1}H NMR (400 MHz, $CDCl_3$) spectrum of hydrogenated cardanol triazole zinc porphyrin

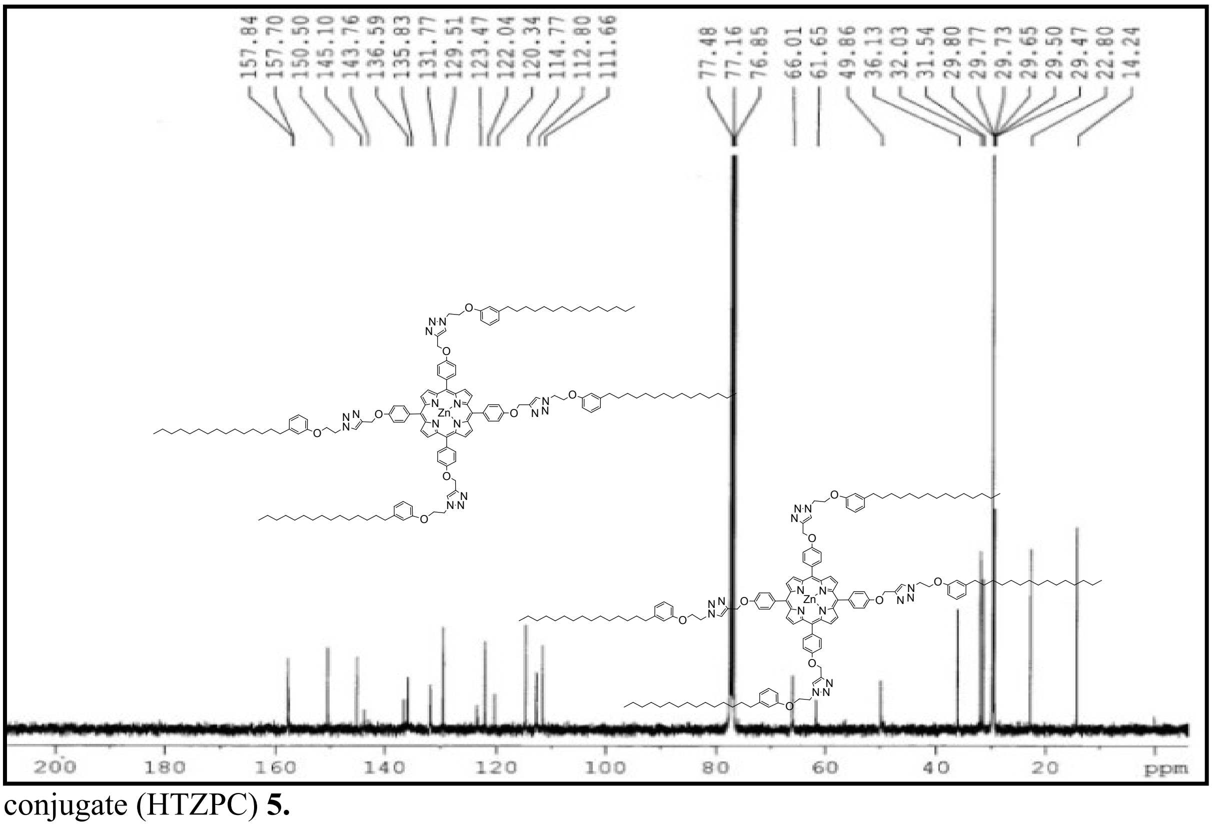

conjugate (HTZPC) **5.**

.

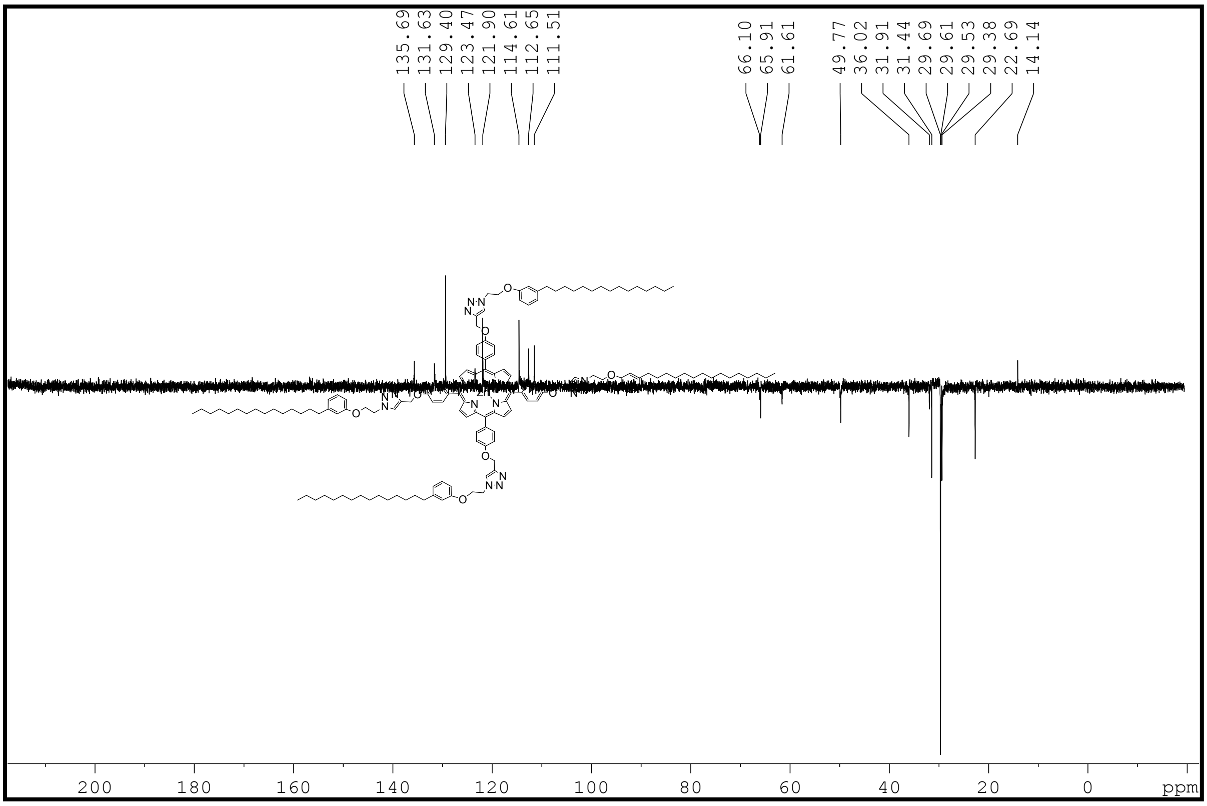

^{13}C NMR (100 MHz, $CDCl_3$) spectrum of hydrogenated cardanol triazole zinc porphyrin conjugate (HTZPC) **5.**

DEPT- 135 NMR spectrum of hydrogenated cardanol triazole zinc porphyrin conjugate (HTZPC) **5.**

.

HRMS spectrum of hydrogenated cardanol triazole zinc porphyrin conjugate (HTZPC) **5.**

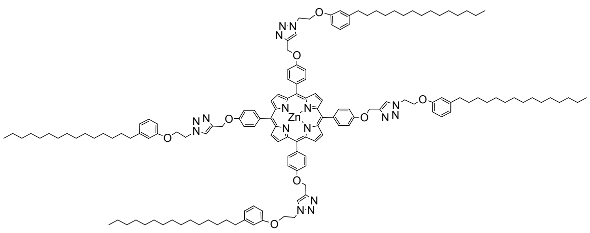

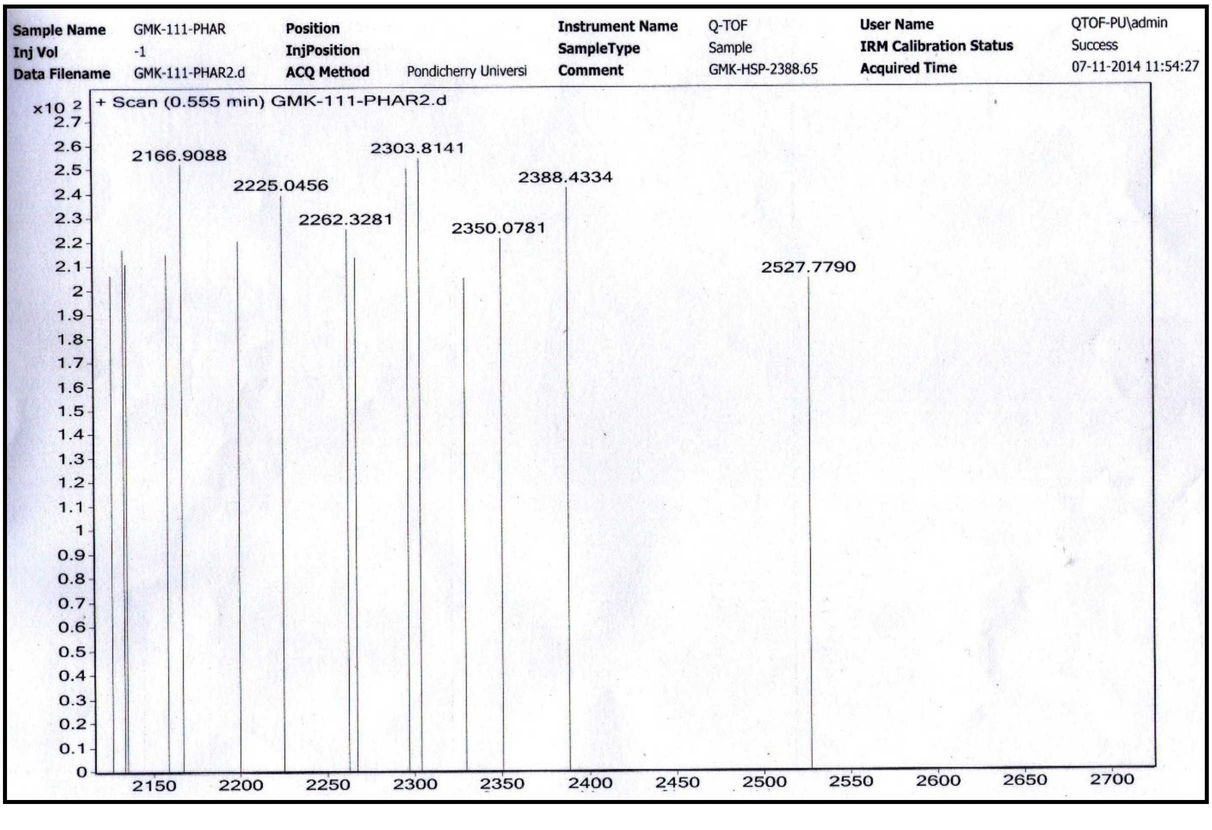

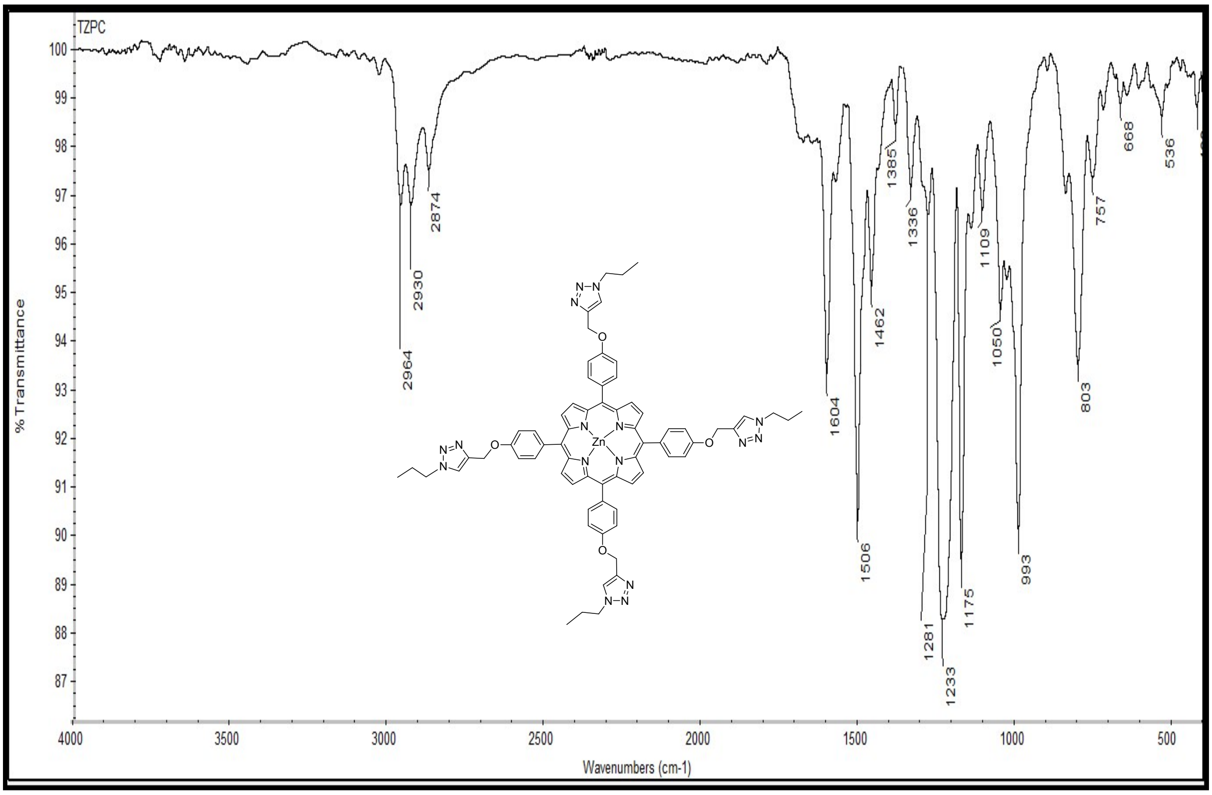

IR (KBr) ν_{max} spectrum of Triazole Zinc Porphyrin Conjugate (TZPC) **6.**

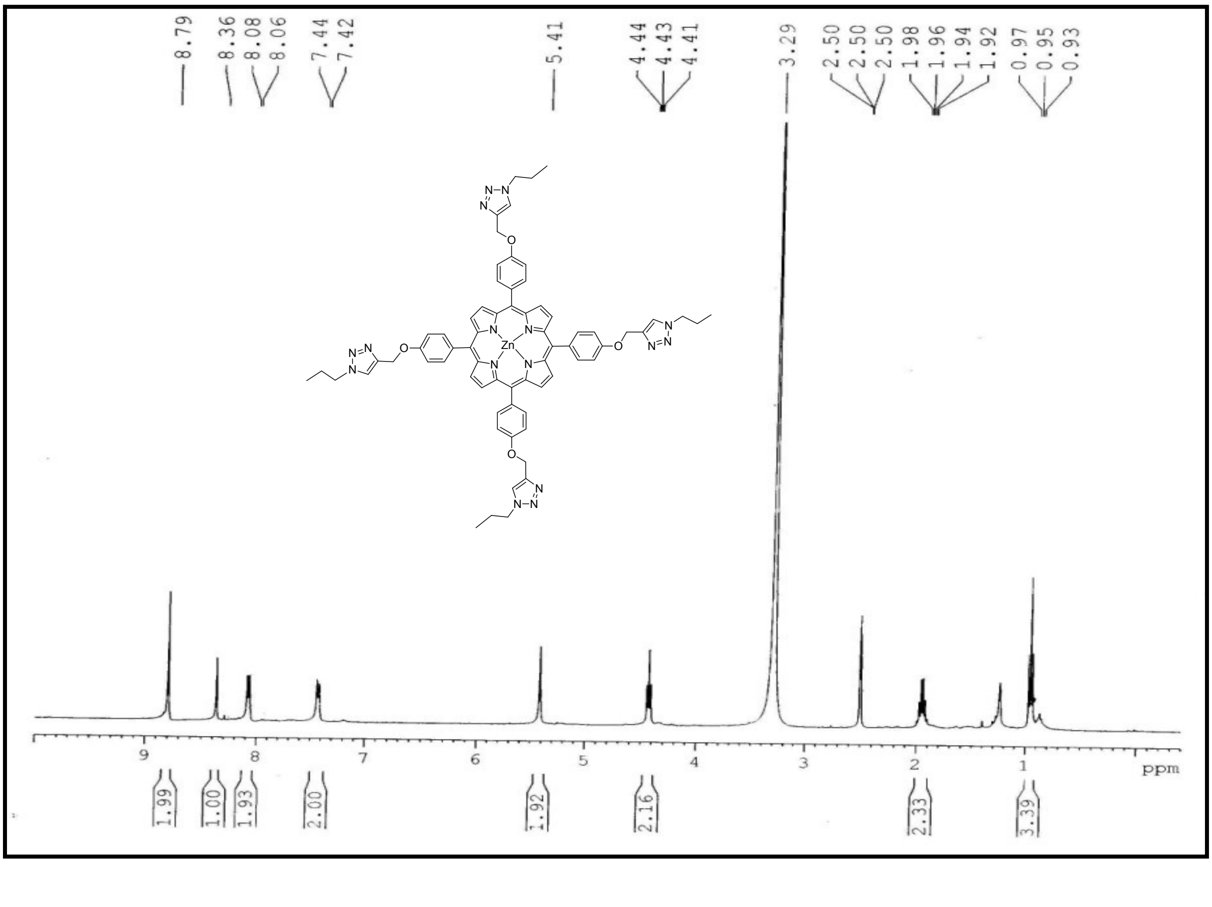

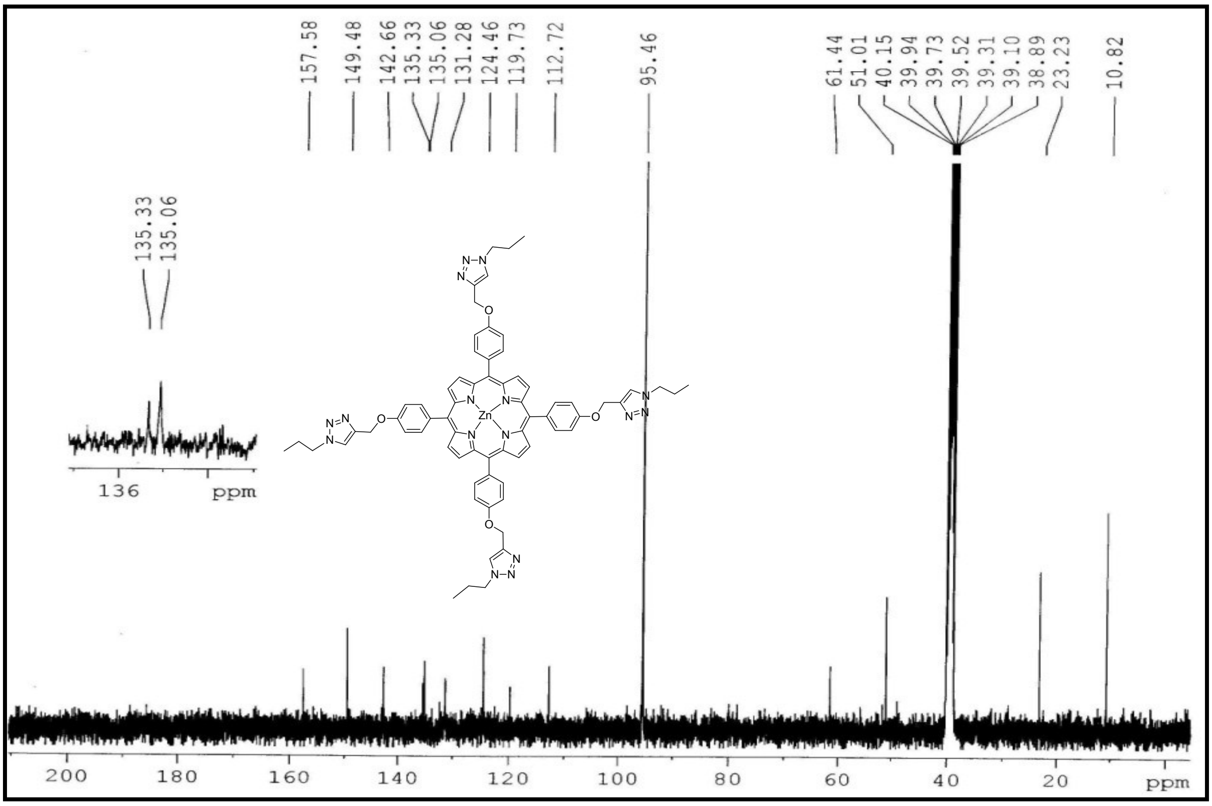

^{1}H NMR (400 MHz, DMSO - d_6) spectrum of Triazole Zinc Porphyrin Conjugate (TZPC) **6**.

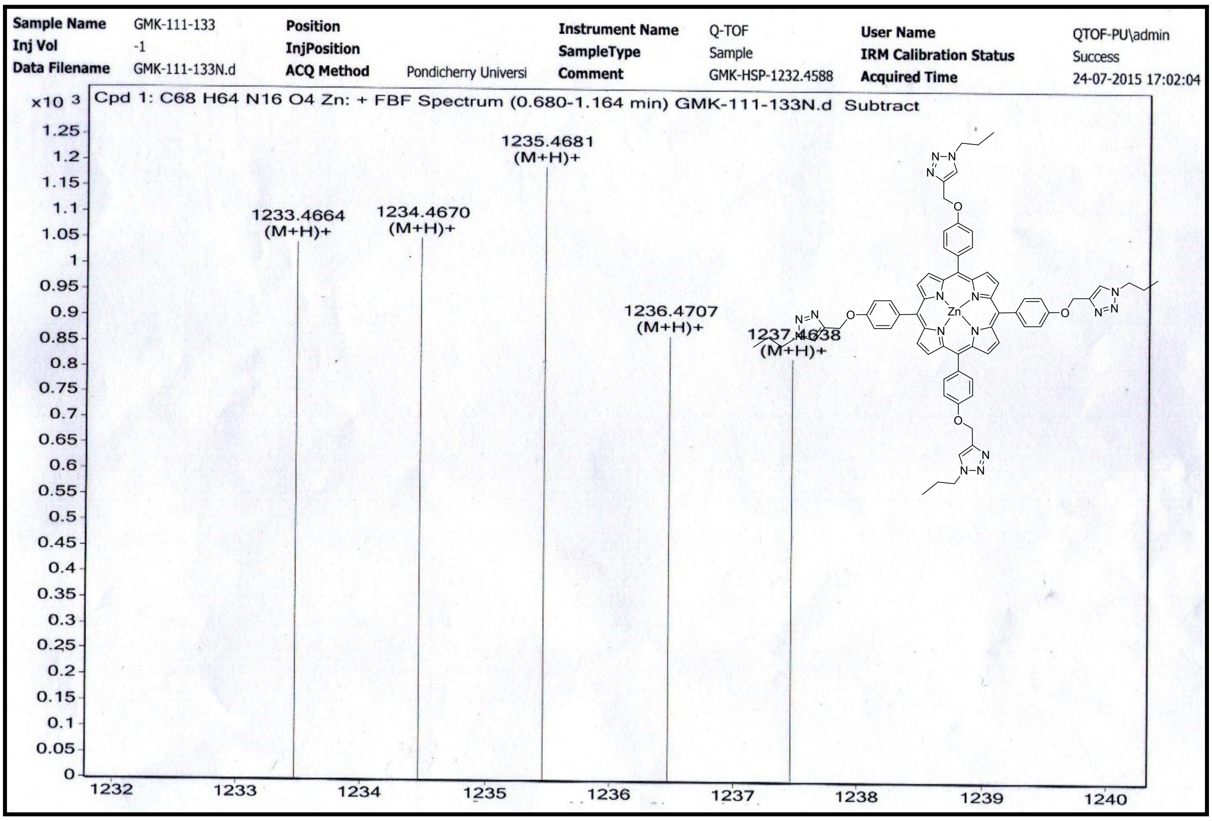

^{13}C NMR (100 MHz, DMSO - d_6) spectrum of Triazole Zinc Porphyrin Conjugate (TZPC) **6.**

HRMS spectrum of Triazole Zinc Porphyrin Conjugate (TZPC) **6.**

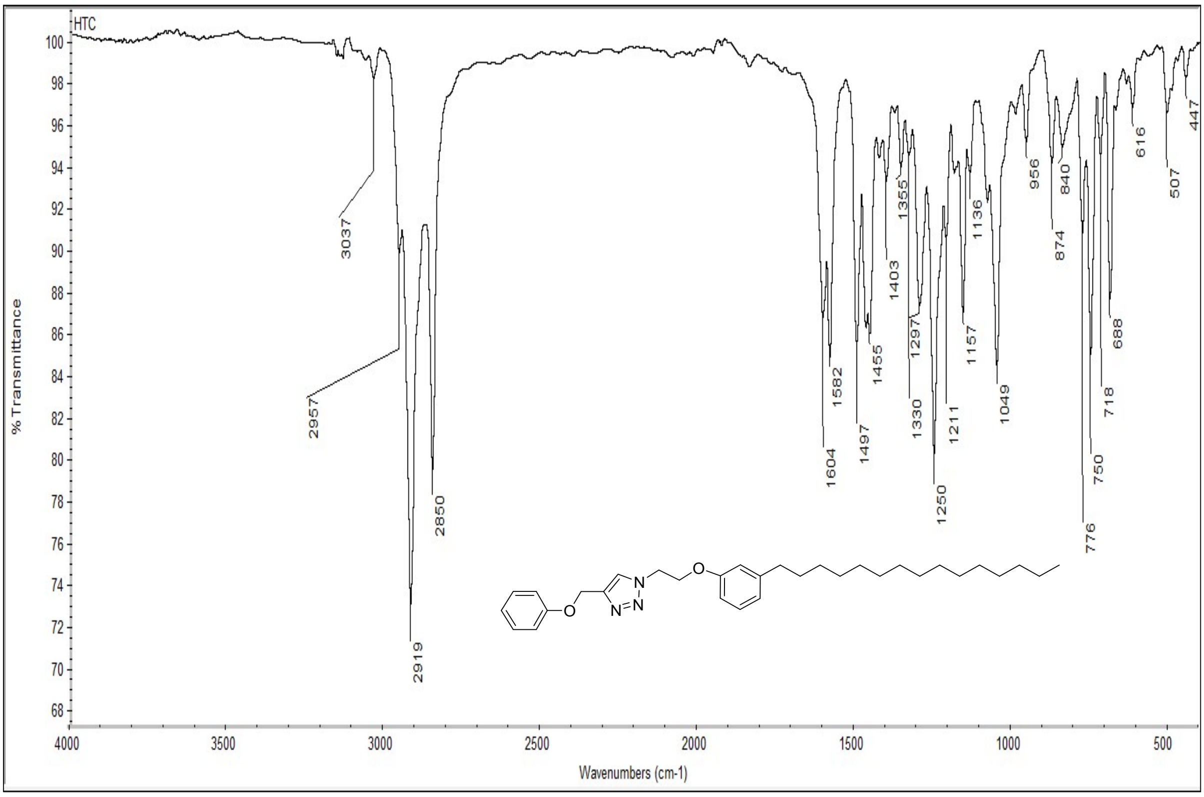

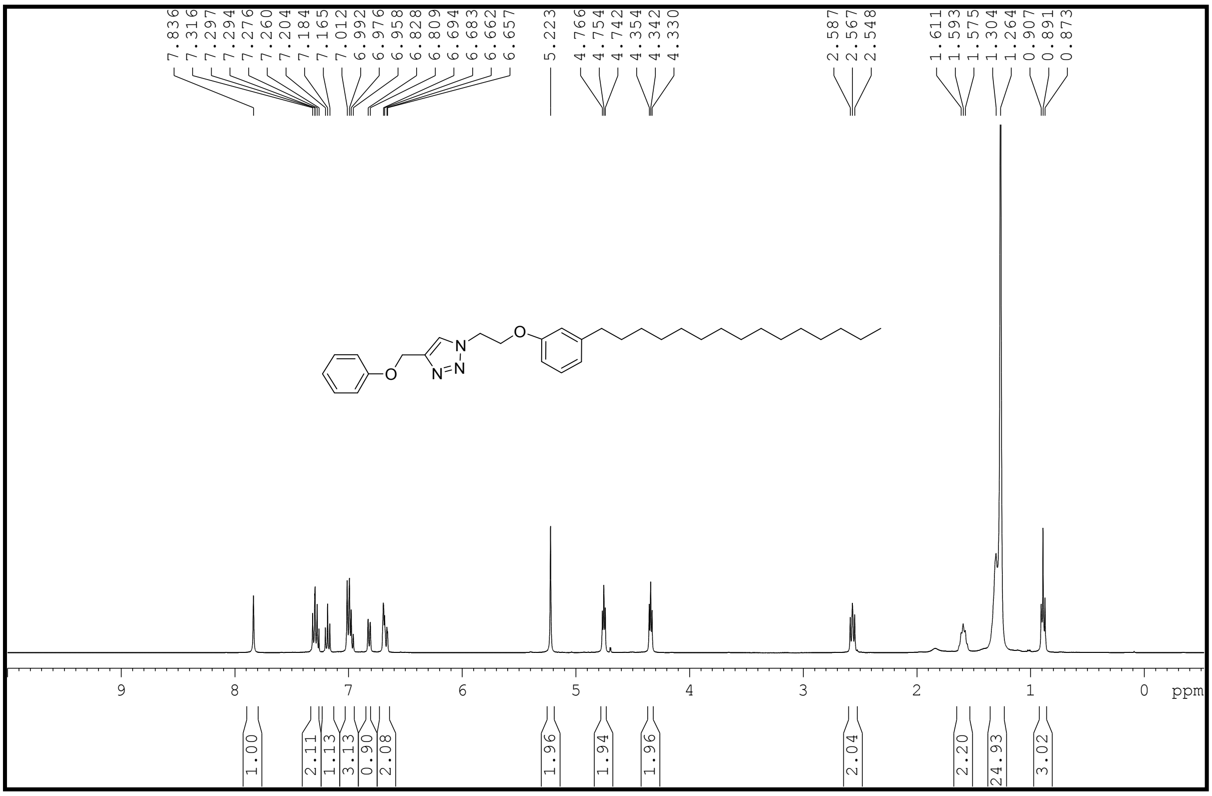

IR (KBr) ν_{max} spectrum of hydrogenated cardanol triazole conjugate (HTC) **8**

^{1}H NMR (400 MHz, $CDCl_3$) spectrum of hydrogenated cardanol triazole conjugate (HTC) **8.**

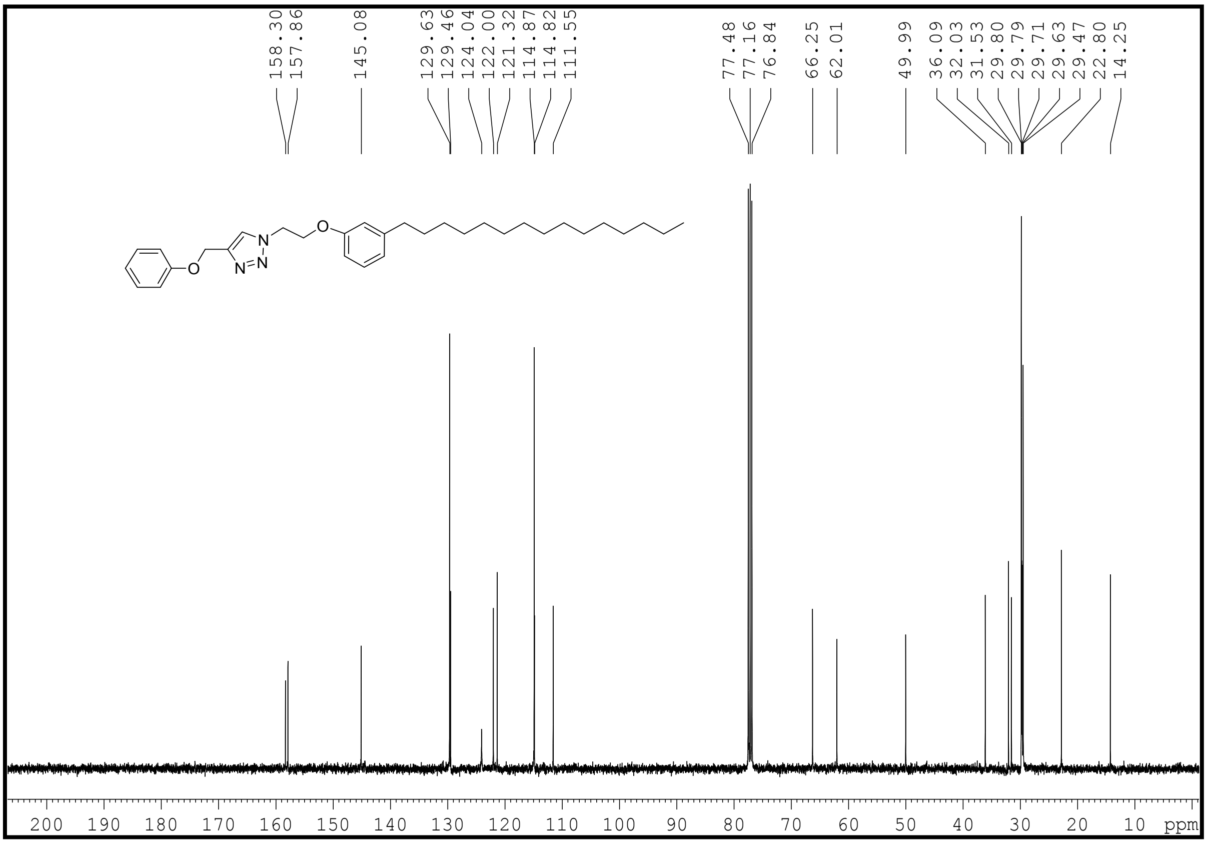

^{13}C NMR (100 MHz, $CDCl_3$) spectrum of hydrogenated cardanol triazole conjugate (HTC) **8.**

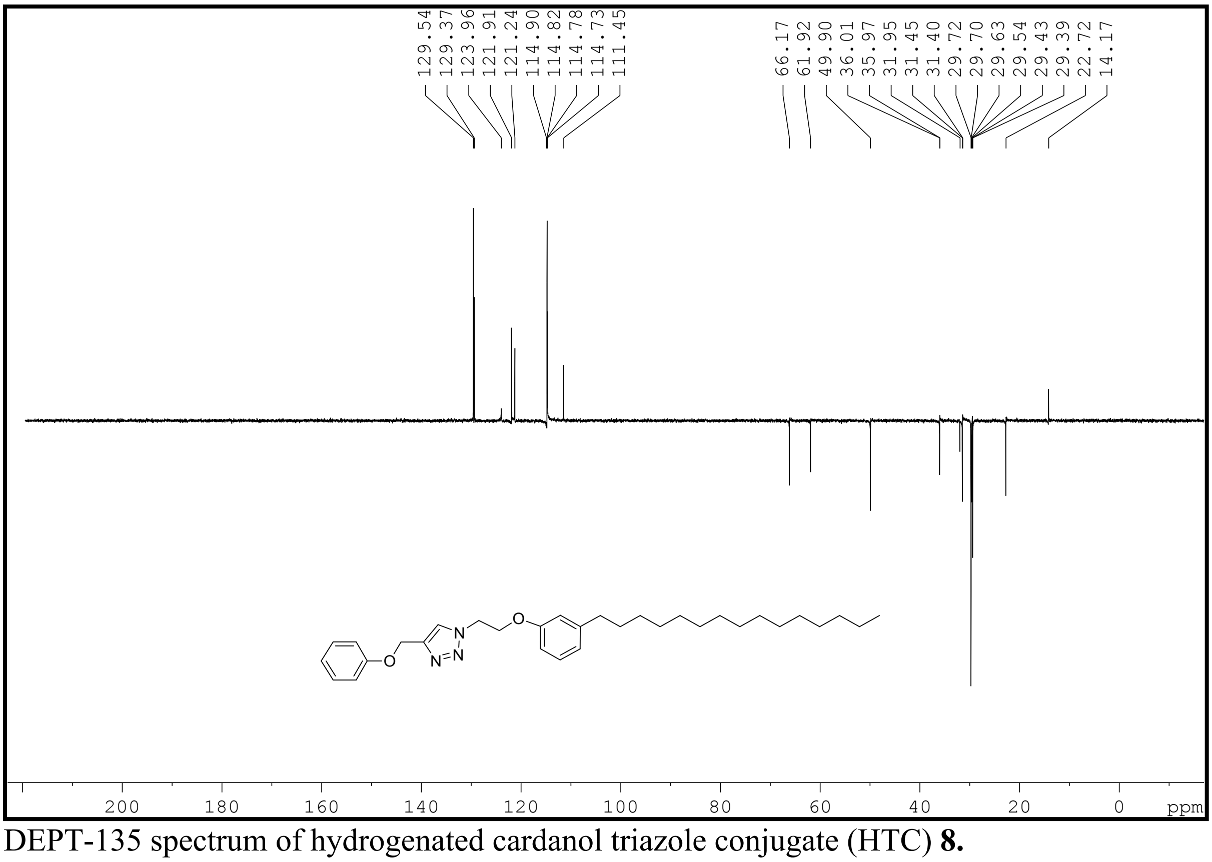

DEPT-135 spectrum of hydrogenated cardanol triazole conjugate (HTC) **8.**

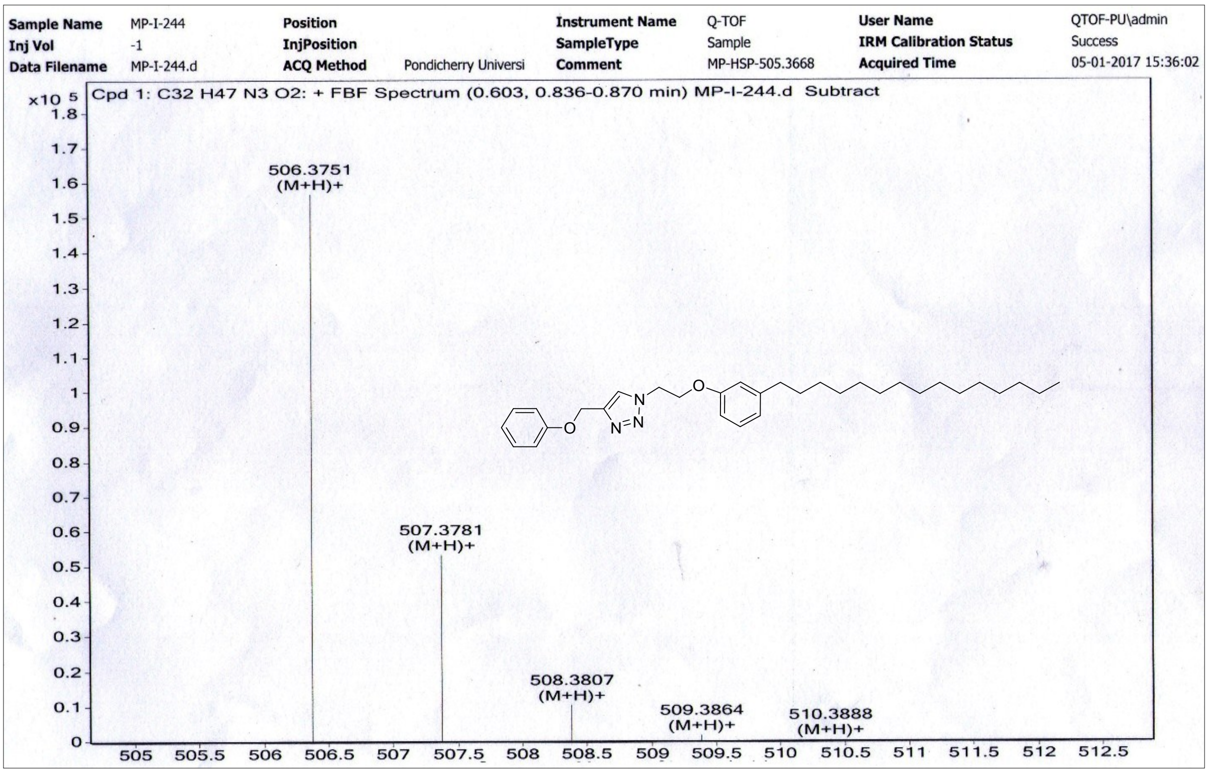

HRMS spectrum of hydrogenated cardanol triazole conjugate (HTC) **8**
